# Grief‐Related Chest Pain: A Review, Conceptual Analysis, and Integrative Model

**DOI:** 10.1111/psyp.70248

**Published:** 2026-01-29

**Authors:** Sophia R. Evstigneev, Frank H. Wilhelm, George M. Slavich, David Blum, Annina Seiler

**Affiliations:** ^1^ Department of Radiation Oncology and Competence Center for Palliative Care University Hospital Zurich and University of Zurich Zürich Switzerland; ^2^ Division of Clinical Psychology and Psychopathology, Department of Psychology University of Salzburg Salzburg Austria; ^3^ Department of Psychiatry and Biobehavioral Sciences University of California Los Angeles California USA

**Keywords:** bereavement, chest pain, grief, psychoneuroimmunology, psychophysiology, Takotsubo syndrome

## Abstract

Although the death of a loved one is a ubiquitous experience with chest pain a commonly reported symptom, grief‐related chest pain and particularly its physiological mechanisms remain under‐investigated. To address this gap, we adopted Rodger's approach to concept analysis to explore the psychoneuroimmunological mechanisms potentially linking bereavement to chest pain and subsequent health outcomes. A PubMed search, followed by a systematic review of existing literature and clinical observations, yielded 220 articles, of which 49 were included in the conceptual analysis. Notably, only four empirical studies specifically examined grief‐related chest pain, but without underlying physiological mechanisms, while 45 studies explored psychoneuroimmune processes more broadly in the context of loss, grief, and bereavement. Based on these findings, we propose a *theoretical model of grief‐related chest pain*. The model integrates insights from studies on autonomic, hemodynamic, musculoskeletal, respiratory, neuroendocrine, and immune changes during grief. It summarizes antecedents, attributes, and consequences of grief‐related chest pain, highlighting the putative interrelated roles of physiological, neuroendocrine, and immune pathways. Our model suggests that grief‐related chest pain may constitute a key physical symptom of grief, arising from physiological responses to acute emotional distress and loss. A deeper understanding of the psychobiological mechanisms underlying this phenomenon may provide prognostic insights, inform disease prevention, improve patient care, and guide the development of targeted interventions. Building on this perspective, we also propose a toolkit to facilitate the assessment of grief‐related chest pain in future empirical studies.

## Impact Statement

We propose that stress‐induced dysregulation of the autonomic, immune, and neuroendocrine systems may contribute to hemodynamic and respiratory changes, potentially leading to grief‐related chest pain, a common yet underexplored physical symptom of grief following the death of a loved one. Given the vital role of the cardiovascular and respiratory systems in the chest, we suggest that chest pain serves an evolutionary function, signaling a perceived threat and promoting care‐seeking behaviors to enhance survival during vulnerable and distressing periods.

## 1 Introduction

The death of a significant person is a profoundly impactful life event that places individuals at increased risk for morbidity and mortality, with cardiovascular disease, including coronary heart disease and stroke, accounting for the largest proportion of these deaths (Kaprio et al. 1987). Chest pain, although often regarded as a hallmark symptom of cardiac stress or cardiovascular disorders, can also present as a manifestation of significant emotional distress (Buckley et al. 2011; Carey et al. 2014; Fagundes and Wu 2020). In the days and weeks following a loss, pain throughout the body (Bradbeer et al. 2003; Seiler et al. 2018; Stroebe et al. 2007), as well as in the chest (Azeez et al. 2022; Granek et al. 2017; Nordström et al. 2024; Spillane et al. 2018), is frequently reported. In some cases, this pain persists for months (Spillane et al. 2018) or even years (Nordström et al. 2024) after the loss. Symptoms in the chest are often described as tightening, aching, or dull pain (Bradbeer et al. 2003; Seiler et al. 2018; Stroebe et al. 2007). Although feelings of numbness, disbelief, sadness, and despair are commonly associated with the loss of a loved one (Fagundes et al. 2018), grief can also manifest with physical symptoms. Chest pain is frequently reported following a significant loss, yet this phenomenon remains empirically under‐investigated. A previous review has addressed the broader relationship between chronic pain and grief (Garciandia Imaz and Rozo Reyes 2019), but to date, no review has specifically examined grief‐related chest pain or synthesized evidence on its potential physiological mechanisms. Given the prevalence of grief in the human experience, further research is warranted to better understand the manifestation of chest pain following a loss, its underlying autonomic, hemodynamic, musculoskeletal, respiratory, neuroendocrine, and immune mechanisms, and health implications.

This review and conceptual analysis aims to advance the literature and understanding of grief‐related chest pain by: (1) distinguishing subclinical grief‐related chest pain from clinical cardiac syndromes such as Takotsubo; (2) proposing a structured framework for its definition and measurement; (3) outlining putative physiological mechanisms and individual‐level moderators (e.g., gender and racial differences, lifetime stress); and (4) presenting a theoretical model of grief‐related chest pain. Together, these contributions move the field beyond descriptive associations and toward a testable, integrative psychophysiological model.

### 1.1 Chest Pain Following Interpersonal Loss

Prior research has explored the general, nonspecific pain experienced by bereaved individuals compared to nonbereaved individuals. For instance, a review by Stroebe et al. (2007) concluded that bereaved people had a greater incidence of physical health complaints, including chest pain, compared to controls. However, no information regarding the temporal occurrence of the chest pain was provided. Furthermore, Bradbeer et al. (2003) found that widowed individuals were three times more likely to report current strong pain when compared to their nonbereaved counterparts. Consistent with these results, Seiler et al. (2018) showed that fatigued bereaved individuals had higher levels of self‐reported pain compared to those in the nonfatigued group. In studies that focused specifically on chest pain, Granek et al. (2017) studied grief symptoms in oncologists following patient death and found that chest pain specifically, in addition to fatigue and general physical discomfort, arose as a key physical symptom. More recently, Nordström et al. (2024) found that bereaved parents and siblings reported somatic symptoms, including pain, and chest pain specifically, up to eight years after a terror attack. Further, in a study by Azeez et al. (2022) on fathers grieving a neonatal death, one father was quoted to say “I feel like every five minutes I'm being stabbed in the chest, you know, because it became a physical, the grief became a physical pain”.

Children have also reported chest pain as part of an emotional response following the death of a loved one. Persson et al. (2021) documented children aged 10–12 years describing chest pain in response to grief, with the study quoting one child after the loss of their grandmother; “I kind of got a heartache then. That someone important in my life was gone”. Early clinical and epidemiological studies have similarly reported associations between bereavement and chest pain. For instance, Beitman et al. (1991) and Crook et al. (1984) described cases of noncardiac chest pain precipitated by intense emotional distress, suggesting a psychogenic or stress‐related component. Roy (1986) and Bradbeer et al. (2003) further noted elevated rates of chest discomfort among bereaved or widowed individuals, even in the absence of cardiac pathology. Collectively, these findings highlight a consistent association between the loss of a loved one and pain, particularly in the chest, across different ages and contexts.

However, despite evidence highlighting the relevance of chest pain in grieving individuals, little is known about the physiological mechanisms through which interpersonal loss may give rise to chest pain. The following sections therefore consider plausible psychophysiological pathways linking grief and stress with pain and, specifically, chest pain.

### 1.2 Psychophysiological Processes Potentially Linking Interpersonal Loss and Chest Pain

Early indicators of possible psychophysiological processes linking interpersonal loss and chest pain come from clinical data on Takotsubo Syndrome. Otherwise commonly known as “broken heart syndrome”, “stress‐induced cardiomyopathy”, “transient apical ballooning syndrome”, “ampulla syndrome”, and “acute stress cardiomyopathy” (Sethi et al. 2023; Singh et al. 2022), Takotsubo Syndrome is a complex and intriguing phenomenon that has been observed following acute physical, but mainly emotional stress, such as the loss of a loved one (Alim et al. 2023; Khalid et al. 2024; Princip et al. 2022; Singh et al. 2022; Templin et al. 2015). First identified in Japan in 1990 by Sato et al. (1990), this syndrome involves left ventricular apical ballooning, mimics myocardial infarction, and is potentially lethal (Butt et al. 2022). Its clinical presentation includes sharp chest pain, dyspnea, ST‐segment alterations shown on electrocardiography (ECG), and cardiac markers indicative of acute coronary syndrome (Merchant et al. 2008). The condition also presents with myocardial macrophage inflammatory infiltrate, altered monocyte subsets, and increased pro‐inflammatory cytokines (Scally et al. 2019). Conversely, on a lesser scale, subclinical chest pain following the death of a loved one may reflect a ubiquitous physical symptom of grief, possibly caused by specific evolutionarily shaped adaptations (O'Connor 2019). It may thus point to intricate links between stress response systems, emotional well‐being, and physical health.

Beyond the clinical presentation of Takotsubo Syndrome, little is known about when and how chest pain manifests following an emotional interpersonal loss and even less is understood about the potential psychophysiological pathways linking acute grief to chest pain. To examine these issues, we conducted a review and conceptual analysis to document what is known about chest pain following loss and to summarize potential mechanisms like chest‐specific sympathetic and parasympathetic autonomic nervous system activity, cardiovascular and respiratory physiology, and neuroendocrine and immunological processes, and how these processes may be linked to the experience of pain in the chest.

### 1.3 Distinguishing Grief‐Related Chest Pain From Cardiac Syndromes

In proposing a working definition of grief‐related chest pain, it is important to distinguish subclinical grief‐related chest pain from clinically relevant chest pain in cardiac syndromes such as Takotsubo Syndrome. Table 1 summarizes the key distinctions between Takotsubo and the subclinical grief‐related chest pain.

**TABLE 1:** Differentiating Takotsubo syndrome and grief‐related chest pain.

Feature	Takotsubo syndrome (“Broken heart syndrome”)	Grief‐related chest pain
Trigger	Emotional, physical, or combined (Templin et al. 2015)	Emotional stress (loss, bereavement, grief‐related events)
Symptoms	Chest pain, dyspnea, syncope, palpitations (Templin et al. 2015)	Subacute/recurrent chest tightness, aching, stabbing, heaviness; may be accompanied by breathlessness or irregular breathing (Azeez et al. 2022; Granek et al. 2017; Nordström et al. 2024; Spillane et al. 2018)
Epidemiology	90% of patients are women (mean age of 67–70) (Templin et al. 2015)	Reported across age and sex groups; prevalence and risk factors not established (Azeez et al. 2022; Nordström et al. 2024; Persson et al. 2021)
Neuroendocrine and immune markers	Troponin, B‐type natriuretic peptide (BNP), N‐terminal proBNP, creatine kinase‐myocardial band (CK‐MB) (Templin et al. 2015) *Emerging markers copeptin and microRNAs (miRNAs) (Budnik et al. 2020; Jaguszewski et al. 2014)	Stress‐related biomarkers of grief (e.g., cortisol, altered cortisol diurnal pattern, inflammatory cytokines such as IL‐6, TNF‐α) (Fagundes et al. 2019; Gerra et al. 2003; Irwin et al. 1988; O'Connor et al. 2012; Seiler et al. 2018), Immune/inflammatory activation (Fagundes et al. 2019; Gerra et al. 2003; Irwin et al. 1988; O'Connor et al. 2012; Seiler et al. 2018) Increased von Willebrand factors (Buckley, Morel‐Kopp, et al. 2012)
ECG	ST‐segment elevation, ST‐segment depression, T‐wave inversion, QTc prolongation (Templin et al. 2015)	To our knowledge, no consistent pathological ECG pattern demonstrated in bereavement‐related, subclinical chest pain. Bereavement studies show increased resting HR and reduced HRV on Holter rather than ischemic ECG changes (Buckley, Stannard, et al. 2012)
Cardiac imaging	Left ventricular dysfunction (hypokinesia, akinesia, or dyskinesia) presenting as apical ballooning or midventricular, basal, or focal wall motion abnormalities (Templin et al. 2015)	Bereavement cohorts rarely include cardiac imaging, direct evidence is limited
Psychophysiology (i.e., autonomic, hemodynamic, musculoskeletal, respiratory)	Stress‐induced catecholamine surge → myocardial stunning (Wittstein et al. 2005)	Stress‐related autonomic dysregulation; increased systolic arterial pressure and systolic pressure load (Buckley et al. 2011; Palitsky et al. 2023), altered breathing patterns (Spillane et al. 2018), muscle tension (Eisenberger et al. 2003; Panksepp 2003; Slavich 2020b)

Abbreviations: BNP, B‐type natriuretic peptide; CK‐MB, creatine kinase–myocardial band; ECG, electrocardiogram; HR, heart rate; HRV, heart rate variability; IL‐6, interleukin‐6; miRNA, microRNA; NT‐proBNP, N‐terminal pro–B‐type natriuretic peptide; QTc, corrected QT interval; SNS, sympathetic nervous system; TNF‐α, tumor necrosis factor alpha.

## 2 Methods

### 2.1 Conceptual Approach

The conceptual analysis involved a structured and thorough examination of the manifestation of chest pain following the death of a significant person, with the aim of clarifying its definition and distinguishing it from related concepts. This paper has employed Rodger's approach to concept analysis, comprised of five distinct steps as seen in Table 2 (Rodgers 2000). This method provides a systematic inductive approach that offers insights, explanations, and descriptions of the concept under investigation (Tofthagen and Fagerstrom 2010). For this article, the model's iterative steps assisted in identifying research and concepts mainly from bereavement literature, psychophysiology, psychoneuroimmunology, psychoneuroendocrinology, and cardiology. The adoption of this methodology has ensured a systematic and comprehensive review of existing research findings related to chest pain in the context of early bereavement. This process can serve to support theory development and facilitate effective communication surrounding the concept of chest pain associated with loss in the early phase of grief.

**TABLE 2:** Phases of Rodger's approach to concept analysis.

Phase	Action
1	Selection of a concept
2	Identify and select an appropriate sample of literature for data collection
3	Identify antecedents, attributes, and consequences of the concept
4	Analyze the data
5	Identify implications for further development of the concept

### 2.2 Literature Search

A comprehensive literature review was conducted on October 20, 2025, using MEDLINE (PubMed). A combination of free text terms including: “bereavement”, “grief”, “chest pain”, “physical symptoms”, “autonomic”, “respiratory”, “neuroendocrine”, “immune”, “hemodynamic”, and “musculoskeletal” encompassed synonyms, abbreviations, and spelling variations. These search terms were selected based on their relevance to the aim of this conceptual analysis and were combined in a way to ensure the chance of finding articles that encompassed the goal of the current paper was maximized and to reduce the risk of missing important publications. Boolean operators were applied to combine individual searches. Inclusion was limited to full‐text articles available in English published in the last ten years and that contained information about the prevalence, etiology, manifestation, or underlying physiological changes following the death of a significant person. Empirical studies from the fields of medicine, biology, psychophysiology, and psychology were considered the most relevant for investigating the concept of grief‐related chest pain. Articles for which only abstracts were available, as well as editorials, conference presentations, and dissertations, were excluded. Articles that solely addressed psychological or physical health outcomes in response to grief but did not explicitly include any self‐report outcomes on pain were excluded. Initially, the literature was screened based on title and abstract relevance, with studies addressing the physiological and psychological correlates which may underlie grief‐related chest pain critically analyzed to identify patterns, gaps, and potential mechanisms underlying this phenomenon. Subsequently, related articles were also extracted in a snowballing system based on the included references. Two independent reviewers (SRE, AS) carefully assessed the selection of all eligible studies. The complete search strategy can be seen in Table 3.

**TABLE 3:** Search strategy and hits.

Search	Search area	Search terms	PubMed (MEDLINE)
#1	Bereavement	“grief”[Title/Abstract] OR “bereavement”[Title/Abstract]	16,069
#2	Chest Pain	(chest pain[Title/Abstract] OR physical symptoms[Title/Abstract] OR physiological[Title/Abstract] OR autonomic[Title/Abstract] OR hemodynamic[Title/Abstract] OR musculoskeletal[Title/Abstract] OR respiratory[Title/Abstract] OR neuroendocrine[Title/Abstract] OR immune[Title/Abstract] OR cardiovascular[Title/Abstract] OR heart rate variability[Title/Abstract] OR blood pressure[Title/Abstract] OR cortisol[Title/Abstract] OR inflammation[Title/Abstract] OR cytokines[Title/Abstract])	4,097,165
#3	Combined searches	#1 AND #2 Filters: English, Full text available, Humans, 2015–2025	220

### 2.3 Literature Synthesis

**FIGURE 1:**
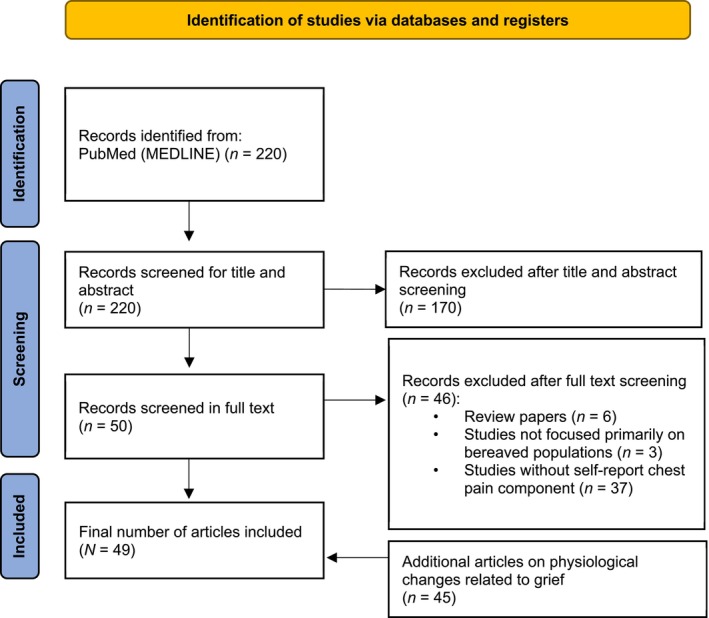
PRISMA flow diagram.

The literature search yielded 220 articles eligible for inclusion into the conceptual analysis. Out of these, four empirical studies included self‐report specifically pertaining to chest pain and physiological measures. A further 45 empirical studies specifically examined the physiological consequences of loss in general. Thus, a total of 49 articles were included. Each of these studies involved bereaved participants, providing direct evidence of autonomic, hemodynamic, musculoskeletal, respiratory, neuroendocrine, or immune changes following spousal, parental, or other close‐person loss. Their inclusion was justified to capture the full spectrum of psychophysiological responses that may be implicated in the onset of grief‐related chest pain, and the potential consequences of this phenomenon. A PRISMA diagram outlining the identification of included articles can be found in Figure 1.

The available data indicate that while pain is a common symptom during bereavement (Bradbeer et al. 2003; Seiler et al. 2018; Stroebe et al. 2007), explicit descriptions of chest pain, particularly during the acute phase of grief, remain scarce in the scientific literature. Consequently, the empirical foundation for understanding grief‐related chest pain is still fragmentary. In this review, we therefore integrate findings from related literatures on grief, stress physiology, and pain to propose a theoretical model outlining the antecedents and physiological attributes of grief‐related chest pain. Finally, we consider the potential clinical and psychosocial consequences of grief‐related chest pain and outline directions for future research.

## 3 Results

### 3.1 Literature Review

The identified literature was stratified according to empirical evidence for three types of grief‐related measurements: (a) self‐reported chest pain (*n* = 4), (b) physiological alterations (*n* = 23), and (c) clinical cardiac outcomes (*n* = 22). Table 4a summarizes studies documenting self‐reported physical manifestations of grief, showing that bereaved individuals frequently describe chest pain and related somatic symptoms as part of their emotional distress following loss. Table 4b presents evidence from studies examining physiological correlates of bereavement, indicating that grief is associated with inflammatory activation, autonomic imbalance, and neuroendocrine dysregulation, processes that may contribute to chest pain and cardiovascular risk. Table 4c compiles studies investigating clinical cardiovascular outcomes after bereavement, demonstrating elevated risks for acute cardiac events, arrhythmias, and long‐term cardiovascular morbidity and mortality following major losses.

**TABLE 4a:** Studies including self‐reported chest pain measurement in bereaved individuals.

Author (Year)	Study population	Self‐report outcomes	Key findings (in relation to grief‐related chest pain)
Azeez et al. (2022)	Bereaved fathers (neonatal death)	Qualitative self‐report including physical symptoms	Fathers described grief manifesting in physical symptoms, including chest pain
Granek et al. (2017)	Oncologists grieving patient deaths	Self‐reported grief and burnout	Physical symptoms of grief included chest and physical discomfort
Nordström et al. (2024)	Parents & siblings bereaved (Utøya)	Somatic and insomnia symptoms	Persistent somatic symptoms including chest pain years postloss
Spillane et al. (2018)	Family members bereaved by suicide	Psychological distress, service use	Reported persistent chest pains, breathlessness and physical pain which endured in the months after deceased's death

Abbreviations: BP, blood pressure; CG, complicated grief; HPA, hypothalamic–pituitary–adrenal (axis); HR, heart rate; HRV, heart rate variability; PGD, prolonged grief disorder; SES, socioeconomic status.

**TABLE 4b:** Studies including physiological measurements in bereaved individuals.

Author (Year)	Study population	Biological or physiological outcomes of interest	Key findings (in relation to grief‐related chest pain)
Brown et al. (2022)	Recently bereaved spouses	Inflammatory cytokines (IL‐6, TNF‐α, CRP)	Grief severity predicted heightened inflammatory reactivity
Buckley et al. (2011)	Recently bereaved spouses	HR, BP, systolic load	Elevated HR and systolic BP in early bereavement
Buckley, Morel‐Kopp, et al. (2012)	Recently bereaved	CRP, vWF, platelet activation	Increased neutrophils, vWF, factor VIII and platelet activation in early bereavement
Buckley, Stannard, et al. (2012)	Bereaved ≤ 6 months	HRV	Reduced HRV (lower vagal tone)
Chen, Suchting, et al. (2023)	Bereaved adults with childhood adversity variation	HRV	Higher HRV mitigated effects of childhood maltreatment on grief, predicting faster recovery among those with adverse childhood experiences
Cohen et al. (2015)	Bereaved adults	CRP, IL‐6	Bereaved adults had increased IL‐6 sE‐selectin. CRP and sICAM‐1 were not elevated vs. nonbereaved
Dietz et al. (2018)	Parentally bereaved vs. nonbereaved youths	Blood pressure recovery to social stress	Bereaved youths showed similar BP reactivity, but slower SBP recovery after social stress among bereaved boys and racial/ethnic minority youths vs. controls
Fagundes et al. (2018)	Recently bereaved spouses	Cytokine production; HRV; distress	Bereavement associated with more pronounced ex vivo pro‐inflammatory cytokine production and lower HRV
Fagundes et al. (2019)	Recently bereaved spouses	IL‐6, TNF‐α, IFN‐γ, IL17‐A, and IL‐2	Those meeting a prespecified grief‐severity cut‐point had higher IFN‐γ, IL‐6, and TNF‐α (T‐cell–derived pro‐inflammatory cytokines)
Fraser et al. (1999)	Recently bereaved adults (spousal loss)	Cortisol, ACTH, catecholamines	HPA and sympathetic activation postloss
Gerra et al. (2003)	Bereaved within 1 year	ACTH, cortisol, catecholamines	Bereaved adults showed increased ACTH, increased cortisol, and DST non suppression
Guevara et al. (2019)	Bereaved vs. controls	Herpesvirus antibody titers	Sex differences in latent herpesvirus (EBV) reactivation: bereaved females showed higher EBV antibody titres; executive inhibition moderated immune dysregulation (especially in men)
Irwin et al. (1988)	Older bereaved adults	NK‐cell activity, lymphocyte counts	Decreased NK‐cell cytotoxicity and increased plasma cortisol in conjugal bereavement vs. controls
LeBlanc et al. (2016)	Adults with Complicated Grief	Autonomic measures (HR, skin conductance)	CG group exhibited attenuated RSA reactivity to some emotional film clips, suggesting blunted PNS reactivity
O'Connor et al. (2012)	Complicated vs. noncomplicated grief	Diurnal cortisol; psychological symptoms	CG associated with a flatter diurnal cortisol slope (HPA dysregulation) vs. non‐CG
Palitsky et al. (2023)	Bereaved adults with PGD	BP, HR, vascular reactivity	Higher PGD symptoms predicted greater SBP reactivity (to grief‐recall)
Paoletti et al. (2023)	Bereaved spouses (SES data)	Ex vivo cytokines + psychological outcomes	Employment/SES differences: bereaved employees (vs retirees) showed higher monocyte‐stimulated IL‐6, TNF‐α, and CCL4 and higher perceived stress
Richardson et al. (2015)	Widowed adults	Diurnal cortisol	In newly bereaved spouses, cortisol levels/slopes varied by death context and gender (prolonged forewarning was linked with higher cortisol, women affected more than men early postloss)
Saavedra Pérez et al. (2017)	Adults 2 years postloss	Salivary cortisol	Complicated grief associated with lower morning cortisol and lower total daily output (chronic‐stress‐like HPA profile) two years postloss
Seiler et al. (2018)	Fatigued bereaved individuals	CRP, IL‐6, TNF‐α + fatigue indices	Higher inflammation (notably CRP) among fatigued bereaved vs. nonfatigued; inflammation correlated with poorer mental health indices
Wu et al. (2021)	Recently bereaved spouses	Inflammation (panel); depressive symptoms	Higher inflammation ~3 months postloss predicted greater depressive symptoms at ~6 months
Wu‐Chung, Brown, et al. (2025)	Recently bereaved spouses	Proinflammatory cytokine production following acute stress	During an acute stress paradigm, widow(er)s showed a steeper IL‐6 increase over time vs. nonbereaved
Wu‐Chung, Kennedy, et al. (2025)	Early widowhood (neuroimaging sample)	Cortical thickness, low‐grade inflammation, cognition	Cortical thickness and inflammation moderated depressive symptom–cognition link in widowhood

Abbreviations: ACTH, adrenocorticotropic hormone; AF, atrial fibrillation; BP, blood pressure; CG, complicated grief; CRP, C‐reactive protein; CV, cardiovascular; HF, heart failure; HPA, hypothalamic–pituitary–adrenal axis; HR, heart rate; HRV, heart rate variability; IL‐6, interleukin‐6; IMT, intima‐media thickness; NK, natural killer (cells); O_2_, oxygen; PGD, prolonged grief disorder; RCT, randomized controlled trial; SES, socioeconomic status; TNF‐α, tumor necrosis factor‐alpha; vWF, von Willebrand factor.

**TABLE 4c:** Studies focusing on clinical cardiovascular outcomes in bereaved individuals.

Author (Year)	Study population	Outcomes measured	Key findings
Chen et al. (2020)	Men with childhood parental death	IHD, stroke	Childhood parental death associated with ~30% increased risk of IHD
Chen, Li, et al. (2022); Chen, Wei, et al. (2022)	Adults with parental death	IHD, stroke	Parental death at younger age associated with ~41% increased risk of IHD and ~30% increased risk of stroke
Chen, Li, et al. (2022); Chen, Wei, et al. (2022)	Heart failure patients	Mortality, rehospitalization	Bereavement worsened HF prognosis
Chen, Janszky, et al. (2023); Chen, Suchting, et al. (2023)	Bereaved in childhood/young adulthood	AF	Bereavement linked to AF onset
Garcia et al. (2025)	Adults who experienced a childhood or adolescent parental death	Cardiovascular disease risk factors	Parental death in childhood/adolescence linked with elevated cardiovascular disease risk in early to mid‐adulthood, with significantly greater risk observed among Black Americans relative to White Americans
Graff et al. (2016)	Adults bereaved of partner	AF incidence	Partner bereavement associated with increased risk of AF within 30 days, peaking at ~8–14 days postloss
Karl et al. (2018)	Newly bereaved adults	Platelet function, BP (low‐dose aspirin trial)	Feasible preventive trial; signaled cardiovascular risk reduction
Kaprio et al. (1987)	Widowed persons	All‐cause and cardiac mortality	Widowhood associated with elevated all‐cause and cardiovascular mortality in the early period after spousal loss
Li et al. (2002)	Parents losing a child	MI incidence	Loss of a child associated with a sharp increase in risk of MI shortly after the event
Lewis et al. (2021)	Midlife women with lifetime losses	Atherosclerosis imaging	Cumulative exposure to upsetting life losses associated with greater carotid intima‐media thickness; association stronger in African‐American women compared to White women, suggesting racial/ethnic disparity in vulnerability
Mostofsky et al. (2012)	Adults reporting bereavement	MI onset	Bereavement associated with an approximately 20‐fold increased risk of MI within first 24 h after loss
Rostila et al. (2013a)	Adults bereaved by sibling death	MI mortality	Sibling bereavement associated with higher risk of fatal MI compared to nonbereaved
Rostila et al. (2013b)	Adults bereaved by sibling death	Fatal stroke	Sibling bereavement predicted increased risk of fatal stroke compared to nonbereaved
Rostila et al. (2015)	Adults bereaved	Mortality and health events following anniversary of loss	Provided evidence of anniversary‐related increases in mortality (“anniversary reaction”)
Sloth et al. (2025)	Widowed adults	Cause‐specific mortality	Indicated a higher risk of death from CVD, digestive diseases, psychiatric diseases or suicide, and respiratory diseases
Tofler et al. (2020)	Bereaved adults	Randomized controlled trial (metoprolol + aspirin)	Cardiovascular risk measures (BP, HR, platelet function)
Wei, Janszky, Fang, et al. (2021); Wei, Janszky, Ljung, et al. (2021); Wei, Olofsson, et al. (2021)	Parents who lost an offspring	Incident ischemic heart disease	Parental bereavement associated with increased IHD risk, highest in early period after loss
Wei, Janszky, et al. (2022)	Bereaved individuals postfirst acute myocardial infarction	Post‐MI prognosis	Prior bereavement before MI associated with worse prognosis (higher rehospitalization/mortality) postfirst acute MI
Wei, Li, Chen, et al. (2022)	Parents who lost a child	Risk of heart failure	Loss of a child associated with increased risk of developing HF in bereaved parent
Wei, Li, Janszky, et al. (2022)	Parents bereaved by child death	Stroke incidence	Child death associated with higher risk of stroke in parents over follow‐up period
Wei et al. (2023)	Parents bereaved by child death	AF risk	Parental bereavement linked with elevated AF risk
Wei et al. (2023)	Individuals bereaved in childhood or young adulthood	AF	Early‐life bereavement (childhood/young adulthood) associated with increased AF risk later in life

Abbreviations: AF, atrial fibrillation; BP, blood pressure; CVD, cardiovascular disease; HF, heart failure; HR, heart rate; IHD, ischemic heart disease; MI, myocardial infarction.

### 3.2 Theoretical Model of Grief‐Related Chest Pain

**FIGURE 2:**
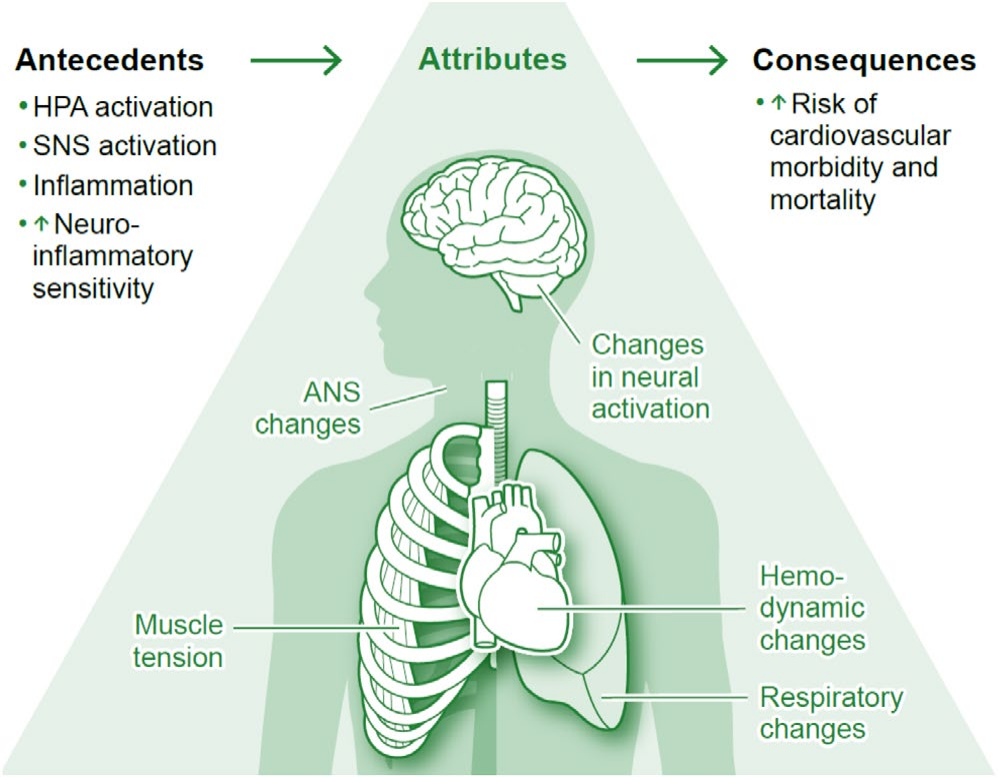
Conceptual model of grief‐related chest pain: antecedents, attributes, and consequences. Antecedents include stress‐induced dysregulation of the autonomic, neuroendocrine, and immune systems, neuroinflammation, and altered neural activation. Attributes compromise autonomic and hemodynamic changes particularly affecting the heart and further include respiratory alterations and increased muscle tension. Consequences include atrial fibrillation, myocardial infarction, and ischemic heart disease that may contribute to an increased risk of cardiovascular morbidity and mortality. The psychophysiological mechanisms delineated in the model are interrelated via multiple pathways: (1) activation of the hypothalamic–pituitary axis (HPA) elevates glucocorticoid levels, suppressing immune function and promoting inflammation; (2) sympathetic nervous system (SNS) activation increases norepinephrine and epinephrine, enhancing heart rate and vasoconstriction; (3) α‐ and β‐adrenergic receptor signaling contributes to myocardial remodeling, dysfunction, and electrical instability; (4) chronic glucocorticoid elevation may further impair cardiovascular function, leading to chest pain and respiratory distress; (5) immune dysfunction characterized by reduced T cells, B cells, and natural killer cell activity, along with increased pro‐inflammatory cytokine signaling (e.g., IFN‐γ, IL‐6, and TNF‐α) not only promotes inflammation but also enhances pain sensitivity. Together, these complex autonomic, neuroendocrine, and neuroimmune interactions may specifically manifest as grief‐related chest pain and cause (sub‐)clinical consequences particularly relating to compromised cardiovascular functioning.

Based on the findings of the literature review and conceptual analysis, we developed a *theoretical model of grief‐related chest pain*, integrating antecedents, defining attributes, and consequences, and highlighting the interrelated roles of neuroendocrine, immune, and psychophysiological pathways in the manifestation of grief‐related chest pain (Figure 2). Following Roger's concept analysis framework, potential antecedents, attributes, and consequences of grief‐related chest pain were derived from the empirical evidence (see Tables 4a, 4b, 4c). This approach helped clarify the conceptual boundaries of chest pain following significant loss and its association with the autonomic, neuroendocrine, and immune systems. Antecedents are described as conditions that may precede the manifestation of, and serve as the prerequisites for, the emergence of grief‐related chest pain. Attributes refer to the distinct characteristics of chest pain, distinguishing it from similar concepts. Consequences are defined as the potential outcomes resulting from grief‐related chest pain. Given the limited empirical evidence on grief‐related chest pain, the model integrates insights from studies on neuroendocrine, immune, and physiological systems, including autonomic, hemodynamic, musculoskeletal, and respiratory changes associated with grief.

In the following, each section begins with a definition of the relevant concept, followed by a synthesis of evidence related to loss, grief, and bereavement, with particular attention to its possible relation with chest pain. Each section concludes with a conceptual integration that summarizes key insights and their implications for understanding grief‐related chest pain.

#### 3.2.1 Potential Antecedents of Grief‐Related Chest Pain

Decades of research demonstrate an intricate connection between emotional states and physical health in general (Slavich and Irwin 2014), with more recent studies highlighting the role of the psychophysiological stress response and systemic inflammation in grief and bereavement (Buckley et al. 2011; Carey et al. 2014; Cohen et al. 2015; Fagundes and Wu 2020). Although the exact physiological origins, or antecedents, of chest pain in this context remain unclear, the field of psychoneuroimmunology has shown how major life stressors, especially experiencing the loss of a loved one, significantly impact the autonomic, neuroendocrine, and immune systems (O'Connor 2019; Seiler et al. 2018, 2020).

#### 3.2.2 Autonomic Nervous System

The autonomic nervous system (ANS), composed of the sympathetic and parasympathetic branches, regulates involuntary mechanisms throughout the body, including the physical response to significant emotional stress (Slavich and Irwin 2014), and may therefore play a crucial role in the onset of chest pain following a loss.

##### 3.2.2.1 Evidence Linking Sympathetic Activation to the Onset of Pain in Grief

In response to a significant emotional loss, the sympathetic nervous system (SNS) becomes overactive and releases catecholamines, including the prominent stress hormones adrenaline (epinephrine) and noradrenalin (norepinephrine). This activation influences cardiovascular tone and immune function, and can promote the release of pro‐inflammatory cytokines (Glaser and Kiecolt‐Glaser 2005). Sustained sympathetic activity may thus contribute to both inflammation and dysregulated stress physiology, processes that have been implicated in pain amplification and cardiovascular reactivity in bereavement.

##### 3.2.2.2 Evidence Linking Sympathetic Activation to Chest Pain

Beyond grief contexts, extensive research demonstrates that sympathetic and β‐adrenergic activation plays a central role in the pathophysiology of both cardiac and noncardiac chest pain. The heart is densely innervated by sympathetic nerves containing α‐ and β‐adrenergic receptors. Acute or sustained activation of adrenergic receptor signaling in response to stress contributes to myocardial remodeling, dysfunction, and electrical instability (Du 2021). Specifically, through its α‐adrenergic signaling, excessive exposure to catecholamines induces elevated aortic diastolic pressure, thereby increasing coronary and cerebral perfusion pressure (Heusch et al. 2000). The α‐adrenergically innervated constriction of coronary arteries can subsequently lead to cardiac ischemia and ischemic chest pain, which may occur when the heart does not receive enough oxygenated blood. Within this context, the role of the SNS may be undermined. In contrast, β‐adrenergic effects increase heart rate, circulatory pressure, and cardiac contractility (Rona 1985). The release of these stress hormones can subsequently promote cardiac repolarization abnormalities (Kivimäki and Steptoe 2018) and, further, induce tachycardia, hypertension, and increased myocardial oxygen demand (Buckley et al. 2011; Fagundes and Wu 2020), which may increase the risk for adverse cardiovascular events, such as arrhythmias (Li et al. 2021; Wittstein et al. 2005). Interestingly but not surprisingly, this increase in catecholamines has also been observed in patients with Takotsubo Syndrome (Barbieri et al. 2021). In cardiovascular medicine, this sympatho‐β‐adrenergic receptor activation is recognized as a hallmark of heart failure (Du 2021).

##### 3.2.2.3 Conceptual Synthesis

Taken together, this evidence suggests that autonomic dysregulation during grief may represent a plausible physiological pathway for grief‐related chest pain. Our theoretical model proposes that prolonged sympathetic activation in response to emotional loss leads to excessive catecholamine release and β‐adrenergic receptor stimulation, increasing cardiac workload, vascular tone, and interoceptive awareness of cardiac sensations. Over time, this sustained activation may interact with inflammatory and musculoskeletal processes, producing recurrent or persistent chest pain in the absence of clinical cardiac pathology.

#### 3.2.3 Neuroendocrine System

Bereavement‐related stress is well known to alter endocrine and immune parameters. For instance, studies have reported elevated cortisol levels, altered diurnal cortisol patterns, and dysregulated immune function in bereaved individuals (Fagundes et al. 2019; Gerra et al. 2003; O'Connor et al. 2012; Seiler et al. 2018). Although these findings do not specifically address chest pain, they suggest that neuroendocrine dysregulation may be an important antecedent of the physiological symptoms experienced during grief.

##### 3.2.3.1 Evidence Linking the Neuroendocrine System to Pain in Grief

Activation of the Hypothalamic–Pituitary–Adrenal (HPA) axis is a hallmark of the stress response and plays a central role in modulating the physiological effects of grief. Emotional stress triggers the release of corticotropin‐releasing hormone (CRH) from the hypothalamus, stimulating adrenocorticotropic hormone (ACTH) release from the pituitary gland and culminating in cortisol secretion from the adrenal cortex (Gerra et al. 2003; Slavich and Irwin 2014). Chronic activation of this system can result in sustained elevations, or in some cases blunted levels, of cortisol, consistent with the physiological “wear and tear” described in the allostatic load model (McEwen 1998; McEwen and Stellar 1993).

Richardson et al. (2015) found that individuals exposed to chronic interpersonal stress displayed a flattened diurnal cortisol rhythm, indicating impaired HPA axis regulation. Similarly, Saavedra Pérez et al. (2017) reported persistent elevations in cortisol levels and disrupted recovery patterns following acute stress in individuals with prolonged grief symptoms, suggesting sustained neuroendocrine arousal. Elevated or dysregulated cortisol levels have been linked to reduced immune competence, systemic inflammation, and cardiovascular (Fraser et al. 1999; Segerstrom and Miller 2004). Such endocrine alterations may heighten bodily arousal and pain sensitivity during bereavement.

##### 3.2.3.2 Evidence Linking the Neuroendocrine System to Chest Pain

Beyond grief, extensive evidence implicates neuroendocrine and HPA axis dysregulation in cardiovascular and chest‐pain‐related outcomes. Cortisol may have debilitating effects on the cardiovascular system, including vasoconstriction, endothelial dysfunction, increasing arterial plaque formation, and higher levels of fibrinogen, thereby enhancing blood clotting, which in turn can lead to chest pain and troubled breathing (Carey et al. 2014; Fraser et al. 1999; Kivimäki and Steptoe 2018). These elevated hemodynamic responses are associated with increased viscosity and subsequently, these responses result in pathophysiological side effects such as electrical instability of the heart, transient myocardial ischemia, plaque disruption, thrombus formation, ventricular fibrillation, myocardial infarction, pulmonary embolism, or stroke (Buckley, Sunari, et al. 2012; Carey et al. 2014; Kivimäki and Steptoe 2018). Moreover, the increase in cortisol and reduced parasympathetic activity can lead to systemic inflammation, which may also serve as an antecedent for chest pain following a loss (Slavich and Irwin 2014).

##### 3.2.3.3 Conceptual Synthesis

Our theoretical model proposes that emotional loss triggers chronic activation, or over time, maladaptive blunting, of the HPA axis, leading to altered cortisol secretion, vascular strain, and inflammatory activation. These processes may amplify cardiovascular sensitivity and interoceptive awareness of pain in the chest region. Through its bidirectional interactions with the autonomic and immune systems, neuroendocrine dysregulation may therefore represent a key antecedent of grief‐related chest pain.

#### 3.2.4 Immune System

##### 3.2.4.1 Evidence Linking the Immune System to Pain in Grief

Although no studies have directly examined immune activation or inflammatory biomarkers in association with chest pain during bereavement, there is an abundance of evidence reporting changes in the immune system following bereavement (Chirinos et al. 2019; Hansel et al. 2010; Knowles et al. 2019; Seiler et al. 2018). Namely, an increased and sustained secretion of pro‐inflammatory cytokines, including interleukin‐1 (IL‐1), interleukin‐6 (IL‐6), and tumor necrosis factor alpha (TNF‐*α*), as well as an impaired immune response manifested by a reduced T‐lymphocyte response and decreased natural killer cell activity, have been found (Gerra et al. 2003; Irwin et al. 1988). Consistent with these findings, Wu et al. (2021) reported that recently bereaved spouses with higher stimulated cytokine production, particularly IL‐6 and TNF‐α, subsequently exhibited greater depressive symptomatology, indicating that immune activation following loss can have sustained psychological and physiological consequences. Complementing these findings, Wu‐Chang et al. (Wu‐Chung, Brown, et al. 2025) reported that individuals exposed to acute psychosocial stress showed heightened cytokine responses (including IL‐6 and IL‐1β reactivity), suggesting that the inflammatory system remains hyperresponsive to stress long after major losses.

IL‐6, a major pro‐inflammatory cytokine secreted in response to infection, trauma, and psychological stress, subsequently stimulates hepatocyte production of C‐reactive protein (CRP), thereby contributing to a nonspecific innate defense mechanism (Cohen et al. 2015; Slavich 2015, 2020a; Vilela and Fontes‐Carvalho 2021). These immune changes indicate sustained activation of inflammatory signaling following loss, though their relationship to chest pain remains to be directly tested.

##### 3.2.4.2 Evidence Linking Inflammation to Bereavement or Stress and Poorer Cardiovascular Outcomes

Evidence increasingly supports inflammation as a mechanism linking bereavement to poorer cardiovascular and physical health outcomes. Inflammation plays a critical role in the onset of cardiovascular disease (CVD) and is strongly associated with cardiovascular mortality (Fagundes et al. 2018) and has been suggested as a key mechanism contributing to the increased risk of CVD in bereaved individuals (Buckley, Morel‐Kopp, et al. 2012; Ridker et al. 1997). Fagundes et al. (2019) hypothesized that younger widows may have a stronger association between grief and inflammation, thereby predisposing them to an increased risk of inflammation following loss of a spouse and subsequently, an increased risk of developing CVD (Fagundes et al. 2019).

Beyond the setting of grief, Tawakol et al. (2017) showed that resting metabolic activity in the amygdala is not only a significant predictor of CVD development (independent of risk factors) but also that activity in this region is associated with perceived stress, increased haemopoietic activity, and increased arterial inflammation (Tawakol et al. 2017). Similarly, studies have linked amygdala activation with inflammatory responses to psychosocial stress (Muscatell et al. 2015, 2016).

##### 3.2.4.3 Conceptual Synthesis

The evidence indicates that bereavement‐related inflammation may represent a key biological mechanism contributing to the experience of grief‐related chest pain. Our theoretical model proposes that the sustained release of pro‐inflammatory cytokines during bereavement heightens systemic inflammation, which in turn sensitizes peripheral nociceptors and central pain pathways, increasing interoceptive awareness of discomfort in the chest region. Chronic inflammatory activity may also exacerbate cardiovascular strain and endothelial dysfunction, creating conditions that mimic cardiac distress. Through its bidirectional interactions with neuroendocrine and autonomic systems, immune dysregulation may thus constitute a central antecedent of grief‐related chest pain.

#### 3.2.5 Neuro‐Immune Cross‐Talk

Although the impact of stress on immune system functioning has been studied extensively, our knowledge of brain‐immune crosstalk, and how brain circuits and sympatho‐adrenergic pathways affect components of the immune system and the body's ability to respond to acute and chronic stressors, remains limited (Bains and Sharkey 2022). Neural–immune signaling involves β‐adrenergic receptor activation within microglia, astrocytes, and dendritic cells, linking emotional and physiological stress to changes in immune responsiveness (Scanzano and Cosentino 2015). Recent research shows that the brain can form neuronal representations of inflammatory information and retrieve them later to reactivate peripheral immune responses (Koren et al. 2021). Consistent with this, Poller et al. (2022) identified specific neural circuits engaged by acute stress that modulate leukocyte activity and disease susceptibility (Poller et al. 2022).

##### 3.2.5.1 Neuro‐Immune Reactivity in Grief

Emerging research indicates that bereavement can heighten neuroimmune reactivity to stress. In an experimental study, Brown et al. (2022) showed that when exposed to acute experimental stressors, bereaved spouses who reported high grief symptoms produced an inflammatory response that is 19% higher compared to bereaved spouses who reported low grief symptoms (Brown et al. 2022). While this study did not assess chest pain specifically, it provides preliminary evidence that grief may sensitize neural–immune pathways implicated in somatic symptoms. Extending these findings, Wu‐Chung, Brown, et al. (2025) and Wu‐Chung, Kennedy, et al. (2025) linked low‐grade inflammation and reduced cortical thickness in emotion‐regulatory brain regions to cognitive impairments in early widowhood, suggesting neuroimmune coupling as a potential mechanism underlying long‐term functional decline (Wu‐Chung, Kennedy, et al. 2025).

##### 3.2.5.2 Neuro‐Immune Sensitivity

Neural‐immune reactivity is not limited to infectious or autoimmune contexts; it has also been observed in situations of social threats, including experiences of loss, social isolation, or rejection (Slavich et al. 2023). Based on the Social Safety Theory by Slavich (2020b, 2022) and Slavich et al. (2023), past experiences of social threat and life adversity can lead to increased neuro‐inflammatory sensitivity. Confronting a new stressor, such as a significant loss, can in turn upregulate neural‐immune reactivity in these individuals, reflected by an enhanced neuroendocrine and immune response, which may be detected in psychophysiological assessments and manifest as pain, and specifically as chest pain. Evidence also indicates that resilient individuals exhibit distinct innate and adaptive immunophenotypes compared to those sensitive to stress (Ahuja et al. 2023). These insights open avenues for future research, such as investigating brain circuits and the role of adrenergic regulation in promoting immune resilience.

##### 3.2.5.3 Evidence Linking Neuro‐Immune Interactions to Pain

Cytokine signaling to the central nervous system (CNS) also plays a crucial role in producing sickness behavior as well as the subjective experience of pain (Kelley et al. 2003; Slavich and Irwin 2014). When pro‐inflammatory cytokines are released, sickness behavior including fatigue, loss of appetite, and social withdrawal are triggered (Kelley et al. 2003; Slavich and Irwin 2014). Further, cytokine signaling enhances sensitivity to pain through the modulation of activity in pain pathways within the CNS, subsequently enhancing the perception of pain (Zhang and An 2007). Therefore, the heightened inflammatory state elicited under chronic stress not only affects the heart in terms of increasing risk for myocardial infarction and CVD (Aalbaek et al. 2017; Buckley et al. 2011) but may also serve as an antecedent for chest pain as a result of localized swelling, increased pressure, and irritation of nociceptors in the chest area.

##### 3.2.5.4 Conceptual Synthesis

Taken together, these findings provide a foundation for understanding how brain–immune interactions could underlie grief‐related somatic symptoms, including chest pain. Our theoretical model proposes that prior experiences of social threat or adversity heighten neuroinflammatory sensitivity through sensitization of central stress circuits and immune signaling pathways. When confronted with a new major loss, these sensitized systems may trigger exaggerated neural–immune responses, reflected in increased neuroendocrine and inflammatory activity, which can manifest as somatic pain, particularly in the chest, where cardiovascular and autonomic pathways converge. This model integrates the Social Safety Theory with psychoneuroimmunological mechanisms (Slavich et al. 2023) to suggest that grief‐related chest pain may represent a psychophysiological expression of neural–immune hyperreactivity.

#### 3.2.6 Potential Attributes of Grief‐Related Chest Pain

##### 3.2.6.1 Autonomic Nervous System and Hemodynamic Changes

Although bereavement is a critical and well‐established risk factor for CVD, and chest pain is one of the most commonly reported somatic symptoms following bereavement (Bradbeer et al. 2003; Nordström et al. 2024), empirical studies have typically focused on general cardiovascular or stress‐related responses rather than chest pain specifically.

##### 3.2.6.2 Evidence on Autonomic and Hemodynamic Changes During Grief

Research on physiological responses to bereavement has demonstrated pronounced autonomic and cardiovascular alterations. Elevated heart rate has been observed in early bereavement (Buckley et al. 2011; Buckley, Stannard, et al. 2012; O'Connor et al. 2002) as well as increased systolic arterial pressure, systolic pressure load (Buckley et al. 2011), slower blood pressure recovery after social stress (Dietz et al. 2018), and reduced heart rate variability (HRV) (Buckley, Stannard, et al. 2012; Fagundes et al. 2018). Heart rate and HRV reflect the balance of sympathetic and parasympathetic activity and are often used as noninvasive measures to quantify cardiac autonomic regulation (Hillebrand et al. 2013). Importantly, lower (high‐frequency) HRV is a strong predictor of cardiovascular events and mortality in patients with coronary artery disease (Tegegne et al. 2023). In addition, bereaved individuals show higher levels of von Willebrand factors, platelets, and granulocyte counts (Buckley, Morel‐Kopp, et al. 2012), consistent with stress‐induced cardiovascular and immune activation. These findings are clinically relevant as hemodynamic and pro‐thrombotic changes are linked to increased cardiovascular risks and mortality (Willeit et al. 2013). However, none of these studies measured concurrent chest pain, leaving the link between autonomic dysregulation and chest discomfort in grief largely inferential.

##### 3.2.6.3 Evidence on Autonomic and Hemodynamic Mechanisms in Chest Pain

Outside bereavement contexts, autonomic and hemodynamic factors are well recognized in the development of noncardiac or stress‐related chest pain. For example, studies of psychogenic chest pain have documented heightened sympathetic activation, reduced vagal tone, and exaggerated cardiovascular reactivity during episodes of discomfort (Beitman et al. 1991; Huffman et al. 2002). Evidence from a systematic review by Forte et al. (2022) further demonstrates that reduced HRV, reflecting diminished parasympathetic modulation, is a robust correlate of heightened pain sensitivity and reduced capacity for physiological regulation across diverse pain conditions (Forte et al. 2022). Adding to this, LeBlanc et al. (2016) found that individuals with complicated grief exhibited an attenuated RSA reactivity to emotional film clips, suggesting a blunted PNS reactivity (LeBlanc et al. 2016). Together, these findings suggest that dysregulated autonomic control and heightened cardiovascular arousal can give rise to chest discomfort even in the absence of cardiac pathology. Such evidence provides a useful comparative framework for understanding how grief‐related autonomic dysregulation might plausibly contribute to the experience of chest pain following loss.

##### 3.2.6.4 Conceptual Synthesis

The literature indicates that bereavement is associated with significant autonomic and hemodynamic changes, yet direct evidence linking these physiological responses to chest pain remains absent. During acute emotional stress, sympathetic activation typically increases heart rate, while chronic stress can lead to persistently elevated resting heart rate and reduced parasympathetic control. Our theoretical model proposes that sustained sympathetic arousal and diminished parasympathetic modulation during grief may heighten cardiovascular reactivity and interoceptive awareness, predisposing individuals to experience chest discomfort.

#### 3.2.7 Musculoskeletal System

To date, no studies have directly investigated musculoskeletal mechanisms underlying chest pain in the context of grief. Although chest pain is frequently reported after the loss of a loved one (Bradbeer et al. 2003; Nordström et al. 2024), the potential contribution of muscle tension or myofascial activation to this experience has not been empirically examined. This absence of evidence highlights an important gap in understanding the somatic substrates of grief‐related chest pain.

##### 3.2.7.1 Evidence on Musculoskeletal Activity and Tension During Emotional Distress

Empirical data on musculoskeletal responses during bereavement are also limited. However, emotional stress more broadly has been associated with increased muscle tension, particularly in the neck, shoulder, and thoracic regions. Electromyography (EMG) provides a reliable physiological measure of such tension and has been validated for detecting stress‐related muscular activation (Lundberg et al. 1994; Pourmohammadi and Maleki 2020), and elevated baseline activity in individuals with anxiety disorders (Pluess et al. 2009). These findings suggest that chronic psychological stress, such as that experienced during bereavement, could manifest in sustained muscular contraction or rigidity.

##### 3.2.7.2 Evidence on Musculoskeletal Mechanisms in Chest Pain

In nonbereavement contexts, musculoskeletal dysfunction is a well‐documented contributor to chest pain. β‐adrenergic receptor signaling mediates SNS activity in skeletal muscle, which under normal conditions promotes beneficial effects on muscle metabolism, function, and mass. Conversely, sustained SNS activity due to chronic stress can have detrimental effects on skeletal muscle, leading to β‐adrenergic receptor desensitization and downregulation, which may exacerbate skeletal myopathy and result in loss of muscle function (Bacurau et al. 2016). Clinically, musculoskeletal chest pain can arise from tension in the intercostal and pectoral muscles, costochondral inflammation, or fascial restriction, all of which can mimic or exacerbate cardiac‐like discomfort. Recent reviews emphasize the need to integrate such peripheral and connective tissue processes into pain research. For instance, Langevin (2024) argues that an excessive focus on neural mechanisms has overshadowed the musculoskeletal and fascial contributions to pain perception (Langevin 2024).

##### 3.2.7.3 Conceptual Synthesis

To our knowledge, muscle tension in the context of bereavement has not yet been described. However, chest pain following a significant loss may have musculoskeletal underpinnings. Our theoretical model proposes that sustained sympathetic activation during bereavement may lead to chronic tension in the intercostal muscles and surrounding fascia, contributing to chest discomfort and labored breathing. Over time, such tension could amplify interoceptive awareness of pain and interact with autonomic and inflammatory pathways described elsewhere in this review. We hypothesize that muscle tension, in particular the chronic tension of the intercostal muscles between the ribs in the chest, may also be correlated to chest pain in the bereaved.

#### 3.2.8 Respiratory System

Evidence directly linking respiratory dysfunction to grief‐related chest pain is limited, but existing studies suggest that respiratory distress frequently cooccurs with chest discomfort following bereavement. Spillane et al. (2018) found that suicide‐bereaved participants reported persistent chest pain, breathlessness, and general physical pain for months after their loss (Spillane et al. 2018). Early reports have also suggested that respiratory irregularities, including sighing respiration, dyspnea, substernal tightness, and palpitations, warrant further investigation in bereaved populations (Crook et al. 1984). Collectively, these findings point to the respiratory system as a potential contributor to the somatic manifestation of grief.

##### 3.2.8.1 Evidence on Respiratory Changes During Emotional Distress

Beyond bereavement‐specific research, studies in affective science have consistently demonstrated that emotional stress and anxiety can produce measurable changes in breathing patterns. Difficulty breathing, labored respiration, or a sense of chest pressure may trigger ineffective or irregular breathing cycles, including hyperventilation. Wilhelm et al. (2006) identified a strong association between fear, anxiety, and respiratory dysregulation, supporting the notion that emotional stress can disrupt normal respiratory control (Wilhelm et al. 2006). Such alterations can increase sympathetic arousal, alter blood gas concentrations, and amplify sensations of chest tightness or discomfort.

Another domain of considerable interest in this realm pertains to the review conducted by Abelson et al. (2010) who investigated the intersection between the HPA axis, respiration, and stress (Abelson et al. 2010). This article suggested that irregular breathing patterns can induce activation of the HPA axis and, further, the HPA axis and respiratory system link to networks involved in emotion processing (Abelson et al. 2010). Additionally, the researchers proposed that there are both afferent and efferent pathways between the hypothalamus in the brain and the respiratory system thereby indicating a bidirectional connection whereby the response to emotional stress, such as a significant loss, can induce respiratory distress, but also vice versa. Considering the involvement of these systems in emotional stress, this explanation of their interactions may at least partially also explain antecedent features of chest pain experienced during grief.

##### 3.2.8.2 Conceptual Synthesis

We propose that emotional stress during bereavement may precipitate irregular breathing patterns and shallow respiration, leading to dyspnea and increased tension in the intercostal and accessory breathing muscles. These biomechanical changes could create a feedback loop in which respiratory discomfort heightens stress and autonomic activation, further perpetuating pain and breathlessness. Through its interactions with the HPA axis and autonomic nervous system, disordered breathing may therefore represent a key pathway linking emotional loss to the subjective experience of chest pain.

#### 3.2.9 Potential Consequences of Grief‐Related Chest Pain

Much evidence indicates an association between experiencing the loss of someone significant and the increased risk of cardiovascular morbidity and mortality, including myocardial infarction (Li et al. 2002; Mostofsky et al. 2012; Rostila et al. 2013b; Wei, Li, Chen, et al. 2022), ischemic heart disease (Chen, Li, et al. 2022; Wei, Janszky, Fang, et al. 2021), and atrial fibrillation (Graff et al. 2016; Wei et al. 2023; Wei, Olofsson, Chen, et al. 2021). These associations were strongest in the weeks and months immediately following the loss but, in some cases, persisted for years. In the same realm, Rostila et al. (2015) demonstrated that bereavement can also produce temporal patterns of heightened health risk, identifying an “anniversary reaction” effect whereby mortality risk temporarily increases around the anniversary of a loved one's death (Rostila et al. 2015).

Whether (nonclinical) chest pain as a sign of an emotional loss may precede the following conditions remains unclear. However, given that chest pain is a standard symptom of the following conditions and there is evidence that bereaved individuals are at a heightened risk for these conditions (Aalbaek et al. 2017; Chen, Janszky, et al. 2023; Chen, Wei, et al. 2022; Graff et al. 2016; Lewis et al. 2021; Mostofsky et al. 2012; Rostila et al. 2013a, 2013b; Sloth et al. 2025; Wei, Janszky, Fang, et al. 2021; Wei et al. 2023; Wei, Li, Chen, et al. 2022; Wei, Li, Janszky, et al. 2022; Wei, Olofsson, Chen, et al. 2021), it is highly likely that grief‐related chest pain may often precede these conditions. Thus, they have been selected for inclusion as potential consequences. In the following section, we highlight some of the most important consequences of stress‐induced cardiovascular events.

##### 3.2.9.1 Atrial Fibrillation

Atrial fibrillation, the most common cardiac arrhythmia, is the leading cardiac cause of stroke and is associated with an increased risk of mortality and CVD, including myocardial infarction and heart failure (Nesheiwat et al. 2024; Wijesurendra and Casadei 2019). Atrial fibrillation is characterized as a tachyarrhythmia and arises due to abnormal electrical activity within the heart, causing subsequent fibrillation (Nesheiwat et al. 2024). Although there are a multitude of risk factors for atrial fibrillation, psychological stress and inflammation have been shown to be associated with an increased risk of atrial fibrillation, with a number of studies indicating the death of a partner, parent, or sibling in either childhood or adulthood is associated with an increased risk of atrial fibrillation (Chen, Janszky, et al. 2023; Graff et al. 2016; Wei et al. 2023; Wei, Olofsson, Chen, et al. 2021). Extending these findings, Sloth et al. (2025) found that bereaved individuals experienced significantly higher mortality within three years of losing a spouse, even after adjusting for sociodemographic and health factors, with the greatest increase in deaths due to cardiovascular causes (Sloth et al. 2025).

##### 3.2.9.2 Myocardial Infarction

Myocardial infarction, often known as a heart attack, occurs as a result of decreased or complete cessation of blood flow to the heart muscle (myocardium) (Ojha and Dhamoon 2024). Following a chronic stressor such as bereavement, pathophysiological changes may lead to an increased risk of myocardial infarction in the following months and years. This association has been observed after the death of a close family member, including both in bereaved children and parents (Li et al. 2002; Rostila et al. 2013b; Wei, Janszky, Ljung, et al. 2022, 2021; Wei, Li, Chen, et al. 2022). One study found an increased risk of myocardial infarction in parents at six years following the loss of a child (Li et al. 2002). Critically, another study found that myocardial infarction had an approximately 20‐fold increased risk of onset within 24 h of the death of a loved one (Mostofsky et al. 2012). Although the incidence rate ratio was shown to decrease each day following the death, it remained elevated for at least one month following the death (Mostofsky et al. 2012). In the first month following bereavement, the occurrence of myocardial infarction was shown to be more than twice as likely for bereaved individuals than their nonbereaved counterparts (Carey et al. 2014). Additionally, parental death has been shown to be associated with a 30% increased risk of myocardial infarction, with the risk being highest in the first three months following the death (Chen, Li, et al. 2022). In turn, Edmondson et al. (2013) published a review discussing the role of emotional stress in the onset of myocardial infarction and reported that an emotional trigger may serve as the final step in a pathophysiological pathway that then induces a heart attack (Edmondson et al. 2013). However, individual differences in vulnerability and risk factors are also emphasized in relation to how an emotional stressor may predict or preclude myocardial infarction.

##### 3.2.9.3 Ischemic Heart Disease

Ischemic heart disease (IHD) is characterized by reduced cardiac blood flow, resulting in unbalanced myocardial oxygen supply and demand (Jensen et al. 2020). Coronary artery disease is the most common underlying pathological process and therefore these terms are often used synonymously (Jensen et al. 2020). One study found that Swedish men who had lost a parent in childhood had an increased risk of IHD (Chen et al. 2020), whereas another study similarly showed that parental death was associated with a 41% increased risk of IHD (Chen, Li, et al. 2022). Wei, Janszky, Fang, et al. (2021); Wei, Janszky, Ljung, et al. (2021); Wei, Olofsson, et al. (2021) corroborated these findings, showing that death of an offspring is associated with heightened risk of IHD (Wei, Janszky, Fang, et al. 2021). The first study to investigate the risk of IHD in the period before the expected death of a spouse indicated that for men only, the risk for hospitalization due to IHD increased 0–3 months prior to the death of a wife, remaining at an elevated risk for up to six months following the loss (Einiö et al. 2017).

## 4 Discussion

### 4.1 Clinical Considerations

There are few experiences more central to the human experience than grief following interpersonal loss. The data available show that although pain is a common symptom during bereavement (Bradbeer et al. 2003; Seiler et al. 2018; Stroebe et al. 2007), descriptions of chest pain during the acute phase of grief remain under‐reported in the scientific literature. This is a critical omission, given the significant increase in morbidity and mortality associated with bereavement, and the weight of CVD in accounting for such morbidities and mortalities. Moreover, although substantial evidence links emotional distress to pain in general, there is limited research specifically on grief‐related chest pain. A comprehensive conceptual analysis that integrates these findings, differentiates grief‐related chest pain from Takotsubo Syndrome, and links them to chest pain, has been lacking. We addressed these issues in the current analysis by distinguishing the ubiquitous phenomenon of chest pain after a loss from more clinical concepts, such as Takotsubo syndrome, ischemia, and myocardial infarction, and by elucidating putative physiological pathways that may link bereavement with chest pain and its subsequent health consequences.

Despite concerns surrounding the declining health reported in individuals following the loss of a loved one, further research is needed to better understand the neuroendocrine, immune, autonomic, hemodynamic, musculoskeletal, and respiratory mechanisms underlying this phenomenon (Ennis and Majid 2021). A systematic review by Ennis and Majid (2021) found that the risk of mortality already increases approximately 30 days prior to spousal loss (where the loss is anticipated), and continues to increase after the loss (Ennis and Majid 2021). The same review proposed that further research is needed on blood pressure, inflammatory changes, and immune system function, with a particular emphasis on the physiological mechanisms of action underlying this increase in mortality risk. The presence of chest pain following a significant emotional loss, in conjunction with knowledge surrounding its onset, duration, intensity, symptoms, and hemodynamic changes, may therefore serve as an important indication or risk factor for subsequent cardiac events, which may in the future be employed as an early warning sign with clinical relevance.

Preliminary intervention studies indicate that early bereavement is a period of heightened but modifiable cardiovascular risk. Karl et al. (2018) found that low‐dose aspirin was a feasible and well‐tolerated preventive strategy, associated with favorable changes in cardiovascular risk markers among recently bereaved individuals (Karl et al. 2018). Similarly, Tofler et al. (2020) showed that short‐term treatment with metoprolol and aspirin attenuated physiological indicators of cardiovascular stress during acute grief (Tofler et al. 2020). Together, these findings indicate that targeted interventions, implemented under medical guidance and supervision, may help mitigate grief‐related cardiovascular activation.

### 4.2 Symptom Perception and Predictive Coding

The concept of pain in a general sense can be viewed as multidimensional, comprised of psychophysiological, psychological (affective and cognitive), ethnological, spiritual, religious, and environmental dimensions (Raja et al. 2020). First and foremost, pain is a subjective experience, and the forms which pain can take are many and complex. Although pain is referred to as an experience coupled with actual or potential tissue damage, pain is also reported where there is no apparent tissue damage or, further, any likelihood of a pathophysiological cause, in which case the pain may be primarily driven by psychological mechanisms. This has also been shown for noncardiac chest pain (Campbell et al. 2017). Affective processes have been shown to profoundly alter pain experiences (Eisenberger et al. 2003), and likely this applies to chest pain.

Importantly, the experience of pain may be shaped or biased by specific cognitive processes, such as predictive coding. Van den Bergh et al. (2017) discuss the concept of predictive coding, highlighting that a contemporary neurocognitive understanding of symptom perception is fundamentally different to the more widely accepted concept that peripheral and physiological changes always precede the perception of physical symptoms (Kolk et al. 2003; Van den Bergh et al. 2017). The authors propose a top‐down processing model to better understand symptom perception, outlining that perception of symptoms in the brain is what influences if and how we experience symptoms. According to this framework, the brain actively predicts sensory input based on previous experiences and expectations, thereby suggesting that the interpretation of physical symptoms, such as chest pain, is a constructional process that is influenced by the constant generation and updating of predictions regarding sensory input in the brain.

Similar to predictive coding, Franke et al. (2022) provide evidence that pain can develop as a classically conditioned response, whereby pain may be triggered by stimuli related to past pain in a psychological trauma context, even without physical harm being present (Franke et al. 2022). Using functional magnetic resonance imaging (fMRI) to examine a neurological pain signature in response to conditioned pain cues, the study showed that brain regions involved in pain perception and emotional memory, namely the anterior cingulate cortex, insula, and amygdala, were particularly active (Franke et al. 2022). This is an interesting and highly relevant finding in the exploration of grief‐related chest pain, considering that previous trauma of grief may condition pain in response to reminders of the loss of a loved one.

### 4.3 Factors Potentially Moderating Chest Pain

Continuing with the notion of pain as a subjective experience, it is important to recognize that several moderating factors likely influence the onset and severity of grief‐related chest pain across individuals.

#### 4.3.1 Context of the Loss

Perhaps most notably, the context or experience of some deaths will inherently be more traumatic than others, such as a young or unexpected death, suicide, or violent death compared to a more natural death. In a study by Groot et al. (2006), the researchers found that those bereaved by suicide reported more pain than those bereaved by natural death, therefore underscoring the importance of psychological trauma related pain and the importance of further investigation into this topic (Groot et al. 2006). Additionally, Richardson et al. (2015) found that bereaved individuals who reported prolonged forewarning of their spouse's death showed higher cortisol levels compared to bereaved individuals without forewarning (Richardson et al. 2015).

#### 4.3.2 Gender

The prevalence of chest pain between sexes must also be considered. Consistent with findings showing that women are more likely to experience severe grief compared to men (Thimm et al. 2020), bereaved women have also been shown to have higher cortisol levels than bereaved men (Richardson et al. 2015). Further, women report more temporary and persistent (general) pain (Crook et al. 1984), and significantly more severe somatic symptoms than men (Nordström et al. 2024). This effect may be explained by the differences between men and women with respect to emotional processing, ANS responses, and hormonal regulation. For example, Wilhelm et al. (2017) investigated sex differences in physiological reactivity to film clips and showed that women exhibited much stronger emotional responses to negative valence films, particularly high‐arousal threat‐related films, when compared to their male counterparts (Wilhelm et al. 2017). The differences were strong and pervasive and included increased heart rate and skin conductance, decreased high‐frequency HRV, increased respiration and respiratory variability, decreased preejection period and pulse transit time, as well as increased facial EMG. This response pattern indicates that women are more likely to experience a stronger activation of the SNS and a less effective PNS counteraction when exposed to threatening scenarios in films.

Adding to this, a novel study by Guevara et al. (2019) found that among bereaved and nonbereaved adults, better executive functioning (a measure of cognitive self‐regulation) was associated with *lower* latent herpesvirus reactivation in men, but this protective association was not evident in women (Guevara et al. 2019). This suggests that men may derive more benefit from cognitive self‐regulation in terms of immune stress‐buffering, whereas women's stress‐immune links may rely on different pathways.

Moreover, hormonal differences may further amplify the variations in response to grief between men and women. Not only does the cortisol and ACTH response to stress in women differ dependent on the menstrual cycle phase (Young and Korszun 2010), but women's cardiovascular systems are also significantly influenced by the sex hormones estrogen and progesterone (Slavich and Sacher 2019). Estrogen has a protective effect on the cardiovascular system by promoting vasodilation and reducing inflammation (Dworatzek and Mahmoodzadeh 2017; Iorga et al. 2017; Mendelsohn and Karas 1999). Dworatzek and Mahmoodzadeh (2017) showed that the role of estrogen in cardiovascular protection was largely responsible for a lower incidence of CVD in premenopausal women compared to men of the same age (Dworatzek and Mahmoodzadeh 2017).

Further, estrogen enhances CRH gene expression and reduces glucocorticoid receptor feedback sensitivity (Tsigos and Chrousos 2002). Therefore, the negative feedback loop which signals for the reduction of cortisol release after stress is less effective in women compared to men (Tsigos and Chrousos 2002). During periods of intense grief, the dysregulation of the HPA axis, resulting in persistently elevated cortisol levels and the potential subsequent suppression of estrogen (Tsigos and Chrousos 2002), may therefore exacerbate the risk of chest pain, inflammation, and other cardiac symptoms. In sum, the increased reporting of more severe somatic symptoms seen in women than men may be explained, at least partially, by the marked difference in emotional processing, ANS response, and hormonal differences between the two sexes. These differences may then subsequently predispose women to be at greater risk of sustained inflammatory, cardiovascular, respiratory, and muscular strain and subsequent chest pain.

#### 4.3.3 Race and Ethnicity

Recent research highlights significant racial and ethnic disparities in the health consequences of bereavement. In a large population‐based study, Garcia et al. (2025) found that the death of a parent was associated with an increased risk of CVD, with the effects disproportionately greater among racially and ethnically minoritized groups compared to White participants (Garcia et al. 2025). These findings suggest that structural inequities and cumulative stress exposures may amplify the physiological toll of bereavement in marginalized populations. Similarly, Lewis et al. (2021) reported that among middle‐aged women, Black participants who experienced a higher cumulative burden of interpersonal losses exhibited greater carotid atherosclerosis than their White counterparts, even after controlling for traditional cardiovascular risk factors (Lewis et al. 2021). Extending this evidence to youth, Dietz et al. (2018) observed that Black bereaved adolescents showed slower systolic blood pressure recovery following social stress compared with White peers, suggesting that racialized stress exposure may compound the physiological burden of bereavement (Dietz et al. 2018). Collectively, these findings highlight that the health toll of bereavement is not evenly distributed, but rather reflects intersecting social, environmental, and racial disparities that shape vulnerability to adverse cardiovascular outcomes. These patterns may likely extend to grief‐related chest pain.

#### 4.3.4 Lifetime Stress, Adverse Events, and Resilience

Lifetime exposure to stress, including early‐life adversity, trauma history, and chronic stress, may strongly shape vulnerability to grief‐related chest pain. Early‐life adversity (e.g., neglect, maltreatment) has enduring effects on stress‐response systems, altering both HPA axis and autonomic regulation. While some individuals exhibit heightened physiological reactivity to stress in adulthood (Shonkoff and Garner 2012), chronic or repeated exposure can also lead to a blunting of these responses, reflecting the physiological “wear and tear” (McEwen 1998; McEwen and Stellar 1993). This cumulative dysregulation of the HPA axis and SNS contributes to maladaptive patterns of cardiovascular and inflammatory activity that may increase susceptibility to stress‐related somatic symptoms, including chest pain during grief. Similarly, trauma history has been linked to exaggerated sympathetic responses and impaired cortisol regulation, which may amplify cardiovascular strain during periods of grief (Heim et al. 2001). Individual differences in baseline autonomic functioning further moderate these effects, as reduced parasympathetic activity and flexibility are associated with prolonged sympathetic activation and greater somatic stress (Thayer and Lane 2000). Importantly, a growing body of research highlights the cumulative role of stress exposure across the life course. Slavich (2016) has proposed that lifetime stress, encompassing early adversity, major traumata, and ongoing stressors, exerts a profound and cumulative influence on health through proinflammatory mechanisms and autonomic dysregulation (Slavich 2016). In line with this, Paoletti et al. (2023) found that among recently widowed adults, those who remained employed had higher perceived stress and elevated monocyte‐stimulated proinflammatory cytokine production compared with their retired peers (Paoletti et al. 2023). Interestingly, within the employed group, higher family income was *associated with* greater stress and grief symptoms, whereas income was unrelated to these outcomes among retired individuals.

In the face of adversity, individuals differ widely in their resilience, with higher resilience associated with more favorable health trajectories (Schafer et al. 2024). Recent research highlights that physiological regulatory capacity, as indexed by HRV, may serve as a key buffer against the long‐term effects of early adversity. Chen, Janszky, et al. (2023); Chen, Suchting, et al. (2023) found that among bereaved older adults, those with histories of childhood maltreatment but higher resting HRV exhibited a steeper decline in grief symptoms over time, indicating faster psychological recovery, whereas individuals with low HRV showed a slower, flatter trajectory of improvement (Chen, Suchting, et al. 2023). These findings suggest that early adversity does not inevitably lead to poorer outcomes; rather, individual differences in physiological self‐regulation can promote adaptive coping and resilience in the face of major life stressors such as bereavement.

#### 4.3.5 Attachment Style

Grief is widely recognized as a fundamental characteristic of most mammals, deeply intertwined with the significance of reproduction, raising offspring, social relationships, and the meaning derived from them (Nesse 2005). From this perspective, the origins of grief symptoms in humans may be traced to the response to loss of a social connection and attachment figure. Humans are intrinsically driven to form and maintain social bonds that are reliable and supportive. The loss of a close relationship disrupts the protective functions of these bonds, leading to profound psychological, behavioral, and somatic reactions (Archer 1999). In this regard, grief‐related chest pain may reflect a secondary outcome of the human capacity for attachment. This raises the possibility that grief‐related chest pain may not always stem from a peripheral physiological cause but may instead manifest as a somatic representation of intense emotional and stress activation from the psychological trauma associated with losing an attachment figure (Khalsa et al. 2018).

On this background, attachment orientation may moderate vulnerability to grief‐related chest pain. Individuals with anxious attachment styles often display heightened physiological reactivity to interpersonal stress, including elevated heart rate, blood pressure, and cortisol responses (Mikulincer and Shaver 2019). In bereavement, this pattern of heightened vigilance and difficulty regulating distress may prolong sympathetic arousal, thereby increasing the likelihood of chest pain. Conversely, avoidantly attached individuals may show blunted physiological responses but may nonetheless be at risk due to chronic suppression of affect and impaired recovery following stress (Diamond and Hicks 2005).

## 5 Limitations

One limitation is the potential for selection bias when identifying and reviewing evidence for antecedents, attributes, and consequences of the construct under study. It is likely that in the coming years the importance of grief‐related chest pain will be more widely recognized, leading to an increase in the number of empirical studies assessing the phenomenology, epidemiology, physiological mechanisms (including both antecedents and concurrent attributes), and health‐related outcomes of chest pain in bereaved individuals in detail. Furthermore, the limited empirical evidence on grief‐related chest pain constrains the validity of our model. Consequently, its interpretation should be approached with caution, and the model may require further refinement as additional evidence becomes available.

## 6 Future Directions

Based on our review, conceptual analysis, and theory on grief‐related chest pain, we suggest that large‐scale longitudinal studies that collect neuroendocrine, immune, hemodynamic, musculoskeletal, and respiratory stress markers are essential to understand the phenomenon of grief‐related chest pain. Such studies would ideally employ multiwave longitudinal designs to capture trajectories of grief‐related symptoms over time, and ecological momentary assessment to provide high‐frequency, real‐world data on chest pain, stress responses, and emotional states (including sadness/grief) in daily life. Priority populations include both younger and older adults, given age‐related differences in cardiovascular vulnerability, as well as individuals experiencing different types of loss (e.g., spousal, parental, child, sudden vs. anticipated loss).

Ideally, such research would include longitudinal health assessments incorporating self‐report measures as well as physiological, neuroendocrine, and immune markers (Mengelkoch, Moriarity, et al. 2023; Moriarity and Slavich 2023, 2024), using a multiomics or comprehensive “stress phenotyping” approach when possible (Gilgoff et al. 2024; Mengelkoch et al. 2024; Mengelkoch, Miryam Schussler‐Fiorenza Rose, et al. 2023). In the context of pain, primarily a subjective and self‐reported phenomenon, the importance of precise assessment by established and validated questionnaires is important. The McGill Pain Questionnaire may be an effective tool in further understanding the complex sensory, affective, and evaluative dimensions of grief‐related chest pain, both in its full‐length (Melzack 1975) and short versions (Lovejoy et al. 2012). The questionnaire, which is designed for pain in a more general sense, combines sensory, affective, and evaluative components to give a highly specified and detailed report of, in this case, chest pain. By combining extensive self‐report data with longitudinal, intensive sampling, and comprehensive physiological assessments, these approaches will help to maximally characterize the biopsychosocial changes that occur during bereavement and that link interpersonal loss with chest pain and subsequent health problems, including increased morbidity and mortality.

## 7 Conclusion

In conclusion, although we have much more to learn about the mechanisms underlying chest pain during the early phase of grief, our review and analysis of the existing literature suggests that loss‐related changes in neuroendocrine, immune, hemodynamic, musculoskeletal, and respiratory pathways are likely involved. Our systematic review and the proposed *theoretical model of grief‐related chest pain* highlight the complexity of potential mechanisms and at the same time the sparsity of empirical data regarding this phenomenon. Looking forward, much more research is needed to provide a clearer and more precise definition of chest pain following grief, to characterize the temporal and severity dynamics of chest (and other) pain symptoms following grief, and to elucidate the biopsychosocial processes linking bereavement, chest pain, health, and mortality risk across different populations.

## Author Contributions

F.H.W. suggested the conceptual idea for this article. The initial draft was written by S.R.E. and subsequently reviewed and edited by A.S., F.H.W., G.M.S., and D.B. All authors read and approved the final version for publication.

## Funding

This study was supported by a grant from the Swiss Cancer League (KLS‐5643‐08‐2022). G.M.S. was supported by grant #OPR21101 from the California Governor's Office of Planning and Research/California Initiative to Advance Precision Medicine. These organizations had no role in planning, writing, editing, or reviewing this article, or in deciding to submit this article for publication.

## Conflicts of Interest

The authors declare no conflicts of interest.

## Data Availability

Data sharing not applicable to this article as no datasets were generated or analyzed during the current study.

## References

[psyp70248-bib-0001] Aalbaek, F. S. , S. Graff , and M. Vestergaard . 2017. “Risk of Stroke After Bereavement—A Systematic Literature Review.” Acta Neurologica Scandinavica 136, no. 4: 293–297. 10.1111/ane.12736. 28220473

[psyp70248-bib-0002] Abelson, J. L. , S. Khan , and N. Giardino . 2010. “HPA Axis, Respiration and the Airways in Stress—A Review in Search of Intersections.” Biological Psychology 84, no. 1: 57–65. 10.1016/j.biopsycho.2010.01.021. 20144683

[psyp70248-bib-0003] Ahuja, S. K. , M. S. Manoharan , G. C. Lee , et al. 2023. “Immune Resilience Despite Inflammatory Stress Promotes Longevity and Favorable Health Outcomes Including Resistance to Infection.” Nature Communications 14, no. 1: 3286. 10.1038/s41467-023-38238-6. PMC10264401 37311745

[psyp70248-bib-0004] Alim, S. , H. Shah , S. Zahera , et al. 2023. “An Update on Takotsubo Syndrome.” Journal of Cardiovascular Medicine (Hagerstown, Md.) 24, no. 10: 691–699. 10.2459/JCM.0000000000001528. 37577868

[psyp70248-bib-0005] Archer, J. 1999. The Nature of Grief: The Evolution and Psychology of Reactions to Loss. Taylor & Frances/Routledge. 10.4324/9780203360651.

[psyp70248-bib-0006] Azeez, S. , K. L. Obst , C. Due , M. Oxlad , and P. Middleton . 2022. “Overwhelming and Unjust: A Qualitative Study of Fathers' Experiences of Grief Following Neonatal Death.” Death Studies 46, no. 6: 1443–1454. 10.1080/07481187.2022.2030431. 35107411

[psyp70248-bib-0007] Bacurau, A. V. , T. F. Cunha , R. W. Souza , V. A. Voltarelli , D. Gabriel‐Costa , and P. C. Brum . 2016. “Aerobic Exercise and Pharmacological Therapies for Skeletal Myopathy in Heart Failure: Similarities and Differences.” Oxidative Medicine and Cellular Longevity 2016: 4374671. 10.1155/2016/4374671. 26904163 PMC4745416

[psyp70248-bib-0008] Bains, J. S. , and K. A. Sharkey . 2022. “Stress and Immunity—The Circuit Makes the Difference.” Nature Immunology 23, no. 8: 1137–1139. 10.1038/s41590-022-01276-1. 35864244

[psyp70248-bib-0009] Barbieri, L. , F. Galli , B. Conconi , et al. 2021. “Takotsubo Syndrome in COVID‐19 Era: Is Psychological Distress the Key?” Journal of Psychosomatic Research 140: 110297. 10.1016/j.jpsychores.2020.110297. 33242703 PMC7666871

[psyp70248-bib-0010] Beitman, B. D. , M. Kushner , and G. T. Grossberg . 1991. “Late Onset Panic Disorder: Evidence From a Study of Patients With Chest Pain and Normal Cardiac Evaluations.” International Journal of Psychiatry in Medicine 21, no. 1: 29–35. 10.2190/0LCN-ETQK-3P3Q-RLVT. 2066255

[psyp70248-bib-0011] Bradbeer, M. , R. D. Helme , H. H. Yong , H. L. Kendig , and S. J. Gibson . 2003. “Widowhood and Other Demographic Associations of Pain in Independent Older People.” Clinical Journal of Pain 19, no. 4: 247–254. 10.1097/00002508-200307000-00008. 12840619

[psyp70248-bib-0012] Brown, R. L. , A. S. LeRoy , M. A. Chen , et al. 2022. “Grief Symptoms Promote Inflammation During Acute Stress Among Bereaved Spouses.” Psychological Science 33, no. 6: 859–873. 10.1177/09567976211059502. 35675903 PMC9343888

[psyp70248-bib-0013] Buckley, T. , A. S. Mihailidou , R. Bartrop , et al. 2011. “Haemodynamic Changes During Early Bereavement: Potential Contribution to Increased Cardiovascular Risk.” Heart, Lung & Circulation 20, no. 2: 91–98. 10.1016/j.hlc.2010.10.073. 21147029

[psyp70248-bib-0014] Buckley, T. , M. C. Morel‐Kopp , C. Ward , et al. 2012. “Inflammatory and Thrombotic Changes in Early Bereavement: A Prospective Evaluation.” European Journal of Preventive Cardiology 19, no. 5: 1145–1152. 10.1177/1741826711421686. 21900365

[psyp70248-bib-0015] Buckley, T. , A. Stannard , R. Bartrop , et al. 2012. “Effect of Early Bereavement on Heart Rate and Heart Rate Variability.” American Journal of Cardiology 110, no. 9: 1378–1383. 10.1016/j.amjcard.2012.06.045. 22853984

[psyp70248-bib-0016] Buckley, T. , D. Sunari , A. Marshall , R. Bartrop , S. McKinley , and G. Tofler . 2012. “Physiological Correlates of Bereavement and the Impact of Bereavement Interventions.” Dialogues in Clinical Neuroscience 14, no. 2: 129–139. 10.31887/DCNS.2012.14.2/tbuckley. 22754285 PMC3384441

[psyp70248-bib-0017] Budnik, M. , S. Bialek , M. Peller , et al. 2020. “Serum Copeptin and Copeptin/NT‐proBNP Ratio—New Tools to Differentiate Takotsubo Syndrome From Acute Myocardial Infarction.” Folia Medica Cracoviensia 60, no. 1: 5–14. 10.24425/fmc.2020.133481. 32658207

[psyp70248-bib-0018] Butt, J. H. , L. E. Bang , R. Rorth , et al. 2022. “Long‐Term Risk of Death and Hospitalization in Patients With Heart Failure and Takotsubo Syndrome: Insights From a Nationwide Cohort.” Journal of Cardiac Failure 28, no. 10: 1534–1544. 10.1016/j.cardfail.2022.02.002. 35167917

[psyp70248-bib-0019] Campbell, K. A. , E. N. Madva , A. C. Villegas , et al. 2017. “Non‐Cardiac Chest Pain: A Review for the Consultation‐Liaison Psychiatrist.” Psychosomatics 58, no. 3: 252–265. 10.1016/j.psym.2016.12.003. 28196622 PMC5526698

[psyp70248-bib-0020] Carey, I. M. , S. M. Shah , S. DeWilde , T. Harris , C. R. Victor , and D. G. Cook . 2014. “Increased Risk of Acute Cardiovascular Events After Partner Bereavement a Matched Cohort Study.” JAMA Internal Medicine 174, no. 4: 598–605. 10.1001/jamainternmed.2013.14558. 24566983

[psyp70248-bib-0021] Chen, H. , T. Hemmingsson , Y. Forsell , M. Rostila , I. Janszky , and K. D. Laszlo . 2020. “Death of a Parent During Childhood and the Risk of Ischemic Heart Disease and Stroke in Adult Men.” Psychosomatic Medicine 82, no. 9: 810–816. 10.1097/PSY.0000000000000861. 32947582

[psyp70248-bib-0022] Chen, H. , I. Janszky , M. Rostila , et al. 2023. “Bereavement in Childhood and Young Adulthood and the Risk of Atrial Fibrillation: A Population‐Based Cohort Study From Denmark and Sweden.” BMC Medicine 21, no. 1: 8. 10.1186/s12916-022-02707-4. 36600284 PMC9814172

[psyp70248-bib-0023] Chen, H. , J. Li , D. Wei , et al. 2022. “Death of a Parent and the Risk of Ischemic Heart Disease and Stroke in Denmark and Sweden.” JAMA Network Open 5, no. 6: e2218178. 10.1001/jamanetworkopen.2022.18178. 35731515 PMC9218848

[psyp70248-bib-0024] Chen, H. , D. Wei , I. Janszky , U. Dahlström , M. Rostila , and K. D. László . 2022. “Bereavement and Prognosis in Heart Failure: A Swedish Cohort Study.” JACC. Heart Failure 10, no. 10: 753–764. 10.1016/j.jchf.2022.05.005. 36175061

[psyp70248-bib-0025] Chen, M. A. , R. Suchting , J. F. Thayer , and C. P. Fagundes . 2023. “Resilience to Stress Across the Lifespan: Childhood Maltreatment, Heart Rate Variability, and Bereavement.” Psychology and Aging 38, no. 3: 247–262. 10.1037/pag0000738. 36951695 PMC10192121

[psyp70248-bib-0026] Chirinos, D. A. , J. C. Ong , L. M. Garcini , D. Alvarado , and C. Fagundes . 2019. “Bereavement, Self‐Reported Sleep Disturbances, and Inflammation: Results From Project HEART.” Psychosomatic Medicine 81, no. 1: 67–73. 10.1097/psy.0000000000000645. 30300238 PMC6309264

[psyp70248-bib-0027] Cohen, M. , S. Granger , and E. Fuller‐Thomson . 2015. “The Association Between Bereavement and Biomarkers of Inflammation.” Behavioral Medicine 41, no. 2: 49–59. 10.1080/08964289.2013.866539. 24266503

[psyp70248-bib-0028] Crook, J. , E. Rideout , and G. Browne . 1984. “The Prevalence of Pain Complaints in a General Population.” Pain 18, no. 3: 299–314. 10.1016/0304-3959(84)90824-8. 6728496

[psyp70248-bib-0029] Diamond, L. M. , and A. M. Hicks . 2005. “Attachment Style, Current Relationship Security, and Negative Emotions: The Mediating Role of Physiological Regulation.” Journal of Social and Personal Relationships 22, no. 4: 499–518. 10.1177/0265407505054520.

[psyp70248-bib-0030] Dietz, L. J. , S. Pham , N. Melhem , G. Porta , and D. A. Brent . 2018. “Blood Pressure Recovery to Social Stress in Parentally Bereaved and Non‐Bereaved Youths.” Journal of Psychosomatic Research 113: 58–65. 10.1016/j.jpsychores.2018.07.016. 30190049 PMC6157912

[psyp70248-bib-0031] Du, X. 2021. “Sympatho‐Adrenergic Mechanisms in Heart Failure: New Insights Into Pathophysiology.” Medical Review 1, no. 1: 47–77. 10.1515/mr-2021-0007. 37724075 PMC10388789

[psyp70248-bib-0032] Dworatzek, E. , and S. Mahmoodzadeh . 2017. “Targeted Basic Research to Highlight the Role of Estrogen and Estrogen Receptors in the Cardiovascular System.” Pharmacological Research 119: 27–35. 10.1016/j.phrs.2017.01.019. 28119050

[psyp70248-bib-0033] Edmondson, D. , J. D. Newman , W. Whang , and K. W. Davidson . 2013. “Emotional Triggers in Myocardial Infarction: Do They Matter?” European Heart Journal 34, no. 4: 300–306. 10.1093/eurheartj/ehs398. 23178642 PMC3549526

[psyp70248-bib-0034] Einiö, E. , H. Moustgaard , P. Martikainen , and T. Leinonen . 2017. “Does the Risk of Hospitalisation for Ischaemic Heart Disease Rise Already Before Widowhood?” Journal of Epidemiology and Community Health 71, no. 6: 599–605. 10.1136/jech-2016-207987. 28235819

[psyp70248-bib-0035] Eisenberger, N. I. , M. D. Lieberman , and K. D. Williams . 2003. “Does Rejection Hurt? An FMRI Study of Social Exclusion.” Science 302, no. 5643: 290–292. 10.1126/science.1089134. 14551436

[psyp70248-bib-0036] Ennis, J. , and U. Majid . 2021. “‘Death From a Broken Heart’: A Systematic Review of the Relationship Between Spousal Bereavement and Physical and Physiological Health Outcomes.” Death Studies 45, no. 7: 538–551. 10.1080/07481187.2019.1661884. 31535594

[psyp70248-bib-0037] Fagundes, C. P. , R. L. Brown , M. A. Chen , et al. 2019. “Grief, Depressive Symptoms, and Inflammation in the Spousally Bereaved.” Psychoneuroendocrinology 100: 190–197. 10.1016/j.psyneuen.2018.10.006. 30368120 PMC6889080

[psyp70248-bib-0038] Fagundes, C. P. , K. W. Murdock , A. LeRoy , F. Baameur , J. F. Thayer , and C. Heijnen . 2018. “Spousal Bereavement Is Associated With More Pronounced Ex Vivo Cytokine Production and Lower Heart Rate Variability: Mechanisms Underlying Cardiovascular Risk?” Psychoneuroendocrinology 93: 65–71. 10.1016/j.psyneuen.2018.04.010. 29702444

[psyp70248-bib-0039] Fagundes, C. P. , and E. L. Wu . 2020. “Matters of the Heart: Grief, Morbidity, and Mortality.” Current Directions in Psychological Science 29, no. 3: 235–241. 10.1177/0963721420917698. 33758475 PMC7983846

[psyp70248-bib-0040] Forte, G. , G. Troisi , M. Pazzaglia , V. Pascalis , and M. Casagrande . 2022. “Heart Rate Variability and Pain: A Systematic Review.” Brain Sciences 12, no. 2: 153. 10.3390/brainsci12020153. 35203917 PMC8870705

[psyp70248-bib-0041] Franke, L. K. , S. F. Miedl , S. K. Danbock , et al. 2022. “Neuroscientific Evidence for Pain Being a Classically Conditioned Response to Trauma‐ and Pain‐Related Cues in Humans.” Pain 163, no. 11: 2118–2137. 10.1097/j.pain.0000000000002621. 35239544

[psyp70248-bib-0042] Fraser, R. , M. C. Ingram , N. H. Anderson , C. Morrison , E. Davies , and J. M. Connell . 1999. “Cortisol Effects on Body Mass, Blood Pressure, and Cholesterol in the General Population.” Hypertension 33, no. 6: 1364–1368. 10.1161/01.hyp.33.6.1364. 10373217

[psyp70248-bib-0043] Garcia, M. A. , B. L. Needham , B. J. Goosby , R. A. Hummer , H. Liu , and D. Umberson . 2025. “Death of a Parent, Racial Inequities, and Cardiovascular Disease Risk in Early toMid‐Adulthood.” Journal of Health and Social Behavior 66, no. 2: 165–181. 10.1177/00221465241273870. 39367799 PMC11971391

[psyp70248-bib-0044] Garciandia Imaz, J. A. , and C. M. Rozo Reyes . 2019. “Chronic Pain and Grief.” Revista Colombiana de Psiquiatría (English ed) 48, no. 3: 182–191. 10.1016/j.rcp.2017.05.008. (Dolor cronico y duelo). 31426921

[psyp70248-bib-0045] Gerra, G. , D. Monti , A. E. Panerai , et al. 2003. “Long‐Term Immune‐Endocrine Effects of Bereavement: Relationships With Anxiety Levels and Mood.” Psychiatry Research 121, no. 2: 145–158. 10.1016/s0165-1781(03)00255-5. 14656449

[psyp70248-bib-0046] Gilgoff, R. , S. Mengelkoch , J. Elbers , et al. 2024. “The Stress Phenotyping Framework: A Multidisciplinary Biobehavioral Approach for Assessing and Therapeutically Targeting Maladaptive Stress Physiology.” Stress 27, no. 1: 2327333. 10.1080/10253890.2024.2327333. 38711299 PMC11219250

[psyp70248-bib-0047] Glaser, R. , and J. K. Kiecolt‐Glaser . 2005. “Stress‐Induced Immune Dysfunction: Implications for Health.” Nature Reviews. Immunology 5, no. 3: 243–251. 10.1038/nri1571. 15738954

[psyp70248-bib-0048] Graff, S. , M. Fenger‐Gron , B. Christensen , et al. 2016. “Long‐Term Risk of Atrial Fibrillation After the Death of a Partner.” Open Heart 3, no. 1: e000367. 10.1136/openhrt-2015-000367. 27099762 PMC4823543

[psyp70248-bib-0049] Granek, L. , M. Ben‐David , S. Shapira , G. Bar‐Sela , and S. Ariad . 2017. “Grief Symptoms and Difficult Patient Loss for Oncologists in Response to Patient Death.” Psycho‐Oncology 26, no. 7: 960–966. 10.1002/pon.4118. 26988940

[psyp70248-bib-0050] Groot, M. H. , J. Keijser , and J. Neeleman . 2006. “Grief Shortly After Suicide and Natural Death: A Comparative Study Among Spouses and First‐Degree Relatives.” Suicide & Life‐Threatening Behavior 36, no. 4: 418–431. 10.1521/suli.2006.36.4.418. 16978096

[psyp70248-bib-0051] Guevara, J. E. , S. Gilbert , K. W. Murdock , R. P. Stowe , and C. P. Fagundes . 2019. “Sex Differences in Executive Functioning and Latent Herpesvirus Reactivation Among Bereaved and Nonbereaved Individuals.” Stress and Health 35, no. 4: 396–406. 10.1002/smi.2867. 30977590 PMC6790147

[psyp70248-bib-0052] Hansel, A. , S. Hong , R. J. Camara , and R. von Kanel . 2010. “Inflammation as a Psychophysiological Biomarker in Chronic Psychosocial Stress.” Neuroscience and Biobehavioral Reviews 35, no. 1: 115–121. 10.1016/j.neubiorev.2009.12.012. 20026349

[psyp70248-bib-0053] Heim, C. , D. J. Newport , R. Bonsall , A. H. Miller , and C. B. Nemeroff . 2001. “Altered Pituitary‐Adrenal Axis Responses to Provocative Challenge Tests in Adult Survivors of Childhood Abuse.” American Journal of Psychiatry 158, no. 4: 575–581. 10.1176/appi.ajp.158.4.575. 11282691

[psyp70248-bib-0054] Heusch, G. , D. Baumgart , P. Camici , et al. 2000. “Alpha‐Adrenergic Coronary Vasoconstriction and Myocardial Ischemia in Humans.” Circulation 101, no. 6: 689–694. 10.1161/01.cir.101.6.689. 10673263

[psyp70248-bib-0055] Hillebrand, S. , K. B. Gast , R. de Mutsert , et al. 2013. “Heart Rate Variability and First Cardiovascular Event in Populations Without Known Cardiovascular Disease: Meta‐Analysis and Dose‐Response Meta‐Regression.” Europace 15, no. 5: 742–749. 10.1093/europace/eus341. 23370966

[psyp70248-bib-0056] Huffman, J. C. , M. H. Pollack , and T. A. Stern . 2002. “Panic Disorder and Chest Pain: Mechanisms, Morbidity, and Management.” Primary Care Companion to the Journal of Clinical Psychiatry 4, no. 2: 54–62. 10.4088/pcc.v04n0203. PMC181226 15014745

[psyp70248-bib-0057] Iorga, A. , C. M. Cunningham , S. Moazeni , G. Ruffenach , S. Umar , and M. Eghbali . 2017. “The Protective Role of Estrogen and Estrogen Receptors in Cardiovascular Disease and the Controversial Use of Estrogen Therapy.” Biology of Sex Differences 8, no. 1: 33. 10.1186/s13293-017-0152-8. 29065927 PMC5655818

[psyp70248-bib-0058] Irwin, M. , M. Daniels , S. C. Risch , E. Bloom , and H. Weiner . 1988. “Plasma Cortisol and Natural Killer Cell Activity During Bereavement.” Biological Psychiatry 24, no. 2: 173–178. 10.1016/0006-3223(88)90272-7. 3390497

[psyp70248-bib-0059] Jaguszewski, M. , J. Osipova , J. R. Ghadri , et al. 2014. “A Signature of Circulating MicroRNAs Differentiates Takotsubo Cardiomyopathy From Acute Myocardial Infarction.” European Heart Journal 35, no. 15: 999–1006. 10.1093/eurheartj/eht392. 24046434 PMC3985061

[psyp70248-bib-0060] Jensen, R. V. , M. V. Hjortbak , and H. E. Botker . 2020. “Ischemic Heart Disease: An Update.” Seminars in Nuclear Medicine 50, no. 3: 195–207. 10.1053/j.semnuclmed.2020.02.007. 32284106

[psyp70248-bib-0061] Kaprio, J. , M. Koskenvuo , and H. Rita . 1987. “Mortality After Bereavement: A Prospective Study of 95,647 Widowed Persons.” American Journal of Public Health 77, no. 3: 283–287. 10.2105/ajph.77.3.283. 3812831 PMC1646890

[psyp70248-bib-0062] Karl, S. , M. Fallon , R. Palitsky , J. A. Martinez , H. Gündel , and M. F. O'Connor . 2018. “Low‐Dose Aspirin for Prevention of Cardiovascular Risk in Bereavement: Results From a Feasibility Study.” Psychotherapy and Psychosomatics 87, no. 2: 112–113. 10.1159/000481862. 29462819

[psyp70248-bib-0063] Kelley, K. W. , R. M. Bluthe , R. Dantzer , et al. 2003. “Cytokine‐Induced Sickness Behavior.” Brain, Behavior, and Immunity 17, no. 1: S112–S118. 10.1016/s0889-1591(02)00077-6. 12615196

[psyp70248-bib-0064] Khalid, N. , S. A. Ahmad , E. Shlofmitz , and L. Chhabra . 2024. Pathophysiology of Takotsubo Syndrome. StatPearls. https://www.ncbi.nlm.nih.gov/pubmed/30844187. 30844187

[psyp70248-bib-0065] Khalsa, S. S. , R. Adolphs , O. G. Cameron , et al. 2018. “Interoception and Mental Health: A Roadmap.” Biological Psychiatry: Cognitive Neuroscience and Neuroimaging 3, no. 6: 501–513. 10.1016/j.bpsc.2017.12.004. 29884281 PMC6054486

[psyp70248-bib-0066] Kivimäki, M. , and A. Steptoe . 2018. “Effects of Stress on the Development and Progression of Cardiovascular Disease.” Nature Reviews Cardiology 15, no. 4: 215–229. 10.1038/nrcardio.2017.189. 29213140

[psyp70248-bib-0067] Knowles, L. M. , J. M. Ruiz , and M. F. O'Connor . 2019. “A Systematic Review of the Association Between Bereavement and Biomarkers of Immune Function.” Psychosomatic Medicine 81, no. 5: 415–433. 10.1097/PSY.0000000000000693. 30950921

[psyp70248-bib-0068] Kolk, A. M. , G. J. Hanewald , S. Schagen , and C. M. van Gijsbers Wijk . 2003. “A Symptom Perception Approach to Common Physical Symptoms.” Social Science & Medicine 57, no. 12: 2343–2354. 10.1016/s0277-9536(02)00451-3. 14572841

[psyp70248-bib-0069] Koren, T. , R. Yifa , M. Amer , et al. 2021. “Insular Cortex Neurons Encode and Retrieve Specific Immune Responses.” Cell 184, no. 25: 6211. 10.1016/j.cell.2021.11.021. 34890554

[psyp70248-bib-0070] Langevin, H. M. 2024. “Addressing Gaps in Pain Research From an Integrated Whole Person Perspective.” Pain 165: S23–S32. 10.1097/j.pain.0000000000003359. 39560412

[psyp70248-bib-0071] LeBlanc, N. J. , L. D. Unger , and R. J. McNally . 2016. “Emotional and Physiological Reactivity in Complicated Grief.” Journal of Affective Disorders 194: 98–104. 10.1016/j.jad.2016.01.024. 26803781

[psyp70248-bib-0072] Lewis, T. T. , M. E. Van Dyke , K. A. Matthews , and E. Barinas‐Mitchell . 2021. “Race/Ethnicity, Cumulative Midlife Loss, and Carotid Atherosclerosis in Middle‐Aged Women.” American Journal of Epidemiology 190, no. 4: 576–587. 10.1093/aje/kwaa213. 33034337 PMC8024052

[psyp70248-bib-0073] Li, J. , D. Hansen , P. B. Mortensen , and J. Olsen . 2002. “Myocardial Infarction in Parents Who Lost a Child: A Nationwide Prospective Cohort Study in Denmark.” Circulation 106, no. 13: 1634–1639. 10.1161/01.cir.0000031569.45667.58. 12270855

[psyp70248-bib-0074] Li, J. , V. K. Somers , X. Gao , et al. 2021. “Evaluation of Optimal Diastolic Blood Pressure Range Among Adults With Treated Systolic Blood Pressure Less Than 130 Mm hg.” JAMA Network Open 4, no. 2: e2037554. 10.1001/jamanetworkopen.2020.37554. 33595663 PMC7890449

[psyp70248-bib-0075] Lovejoy, T. I. , D. C. Turk , and B. J. Morasco . 2012. “Evaluation of the Psychometric Properties of the Revised Short‐Form McGill Pain Questionnaire.” Journal of Pain 13, no. 12: 1250–1257. 10.1016/j.jpain.2012.09.011. 23182230 PMC3513374

[psyp70248-bib-0076] Lundberg, U. , R. Kadefors , B. Melin , et al. 1994. “Psychophysiological Stress and EMG Activity of the Trapezius Muscle.” International Journal of Behavioral Medicine 1, no. 4: 354–370. 10.1207/s15327558ijbm0104_5. 16250795

[psyp70248-bib-0077] McEwen, B. S. 1998. “Protective and Damaging Effects of Stress Mediators.” New England Journal of Medicine 338, no. 3: 171–179. 10.1056/NEJM199801153380307. 9428819

[psyp70248-bib-0078] McEwen, B. S. , and E. Stellar . 1993. “Stress and the Individual. Mechanisms Leading to Disease.” Archives of Internal Medicine 153, no. 18: 2093–2101. 8379800

[psyp70248-bib-0079] Melzack, R. 1975. “The McGill Pain Questionnaire: Major Properties and Scoring Methods.” Pain 1, no. 3: 277–299. 10.1016/0304-3959(75)90044-5. 1235985

[psyp70248-bib-0080] Mendelsohn, M. E. , and R. H. Karas . 1999. “The Protective Effects of Estrogen on the Cardiovascular System.” New England Journal of Medicine 340, no. 23: 1801–1811. 10.1056/NEJM199906103402306. 10362825

[psyp70248-bib-0081] Mengelkoch, S. , J. Gassen , S. Lev‐Ari , et al. 2024. “Multi‐Omics in Stress and Health Research: Study Designs That Will Drive the Field Forward.” Stress 27, no. 1: 2321610. 10.1080/10253890.2024.2321610. 38425100 PMC11216062

[psyp70248-bib-0082] Mengelkoch, S. , S. Miryam Schussler‐Fiorenza Rose , Z. Lautman , et al. 2023. “Multi‐Omics Approaches in Psychoneuroimmunology and Health Research: Conceptual Considerations and Methodological Recommendations.” Brain, Behavior, and Immunity 114: 475–487. 10.1016/j.bbi.2023.07.022. 37543247 PMC11195542

[psyp70248-bib-0083] Mengelkoch, S. , D. P. Moriarity , A. M. Novak , M. P. Snyder , G. M. Slavich , and S. Lev‐Ari . 2023. “Using Ecological Momentary Assessments to Study How Daily Fluctuations in Psychological States Impact Stress, Well‐Being, and Health.” Journal of Clinical Medicine 13, no. 1: 24. 10.3390/jcm13010024. 38202031 PMC10779927

[psyp70248-bib-0084] Merchant, E. E. , S. W. Johnson , P. Nguyen , C. Kang , and W. K. Mallon . 2008. “Takotsubo Cardiomyopathy: A Case Series and Review of the Literature.” Western Journal of Emergency Medicine 9, no. 2: 104–111. https://www.ncbi.nlm.nih.gov/pubmed/19561716. 19561716 PMC2672240

[psyp70248-bib-0085] Mikulincer, M. , and P. R. Shaver . 2019. “Attachment Orientations and Emotion Regulation.” Current Opinion in Psychology 25: 6–10. 10.1016/j.copsyc.2018.02.006. 29494853

[psyp70248-bib-0086] Moriarity, D. P. , and G. M. Slavich . 2023. “The Future Is Dynamic: A Call for Intensive Longitudinal Data in Immunopsychiatry.” Brain, Behavior, and Immunity 112: 118–124. 10.1016/j.bbi.2023.06.002. 37286174 PMC10411233

[psyp70248-bib-0087] Moriarity, D. P. , and G. M. Slavich . 2024. “Toward a Dynamic Immunopsychiatry.” Brain, Behavior, and Immunity 118: 50–51. 10.1016/j.bbi.2024.02.011. 38365011 PMC11225623

[psyp70248-bib-0088] Mostofsky, E. , M. Maclure , J. B. Sherwood , G. H. Tofler , J. E. Muller , and M. A. Mittleman . 2012. “Risk of Acute Myocardial Infarction After the Death of a Significant Person in One's Life: The Determinants of Myocardial Infarction Onset Study.” Circulation 125, no. 3: 491–496. 10.1161/CIRCULATIONAHA.111.061770. 22230481 PMC3397171

[psyp70248-bib-0089] Muscatell, K. A. , K. Dedovic , G. M. Slavich , et al. 2015. “Greater Amygdala Activity and Dorsomedial Prefrontal‐Amygdala Coupling Are Associated With Enhanced Inflammatory Responses to Stress.” Brain, Behavior, and Immunity 43: 46–53. 10.1016/j.bbi.2014.06.201. 25016200 PMC4368432

[psyp70248-bib-0090] Muscatell, K. A. , K. Dedovic , G. M. Slavich , et al. 2016. “Neural Mechanisms Linking Social Status and Inflammatory Responses to Social Stress.” Social Cognitive and Affective Neuroscience 11, no. 6: 915–922. 10.1093/scan/nsw025. 26979965 PMC4884319

[psyp70248-bib-0091] Nesheiwat, Z. , A. Goyal , and M. Jagtap . 2024. Atrial Fibrillation. StatPearls. https://www.ncbi.nlm.nih.gov/pubmed/30252328. 30252328

[psyp70248-bib-0092] Nesse, R. M. 2005. “An Evolutionary Framework for Understanding Grief.” In Spousal Bereavement in Late Life, edited by D. Carr , R. M. Nesse , and C. B. Wortman . Springer Publishing.

[psyp70248-bib-0093] Nordström, E. E. L. , R. Kaltiala , P. Kristensen , and J. C. Thimm . 2024. “Somatic Symptoms and Insomnia Among Bereaved Parents and Siblings Eight Years After the Utøya Terror Attack.” European Journal of Psychotraumatology 15, no. 1: 2300585. 10.1080/20008066.2023.2300585. 38214224 PMC10791101

[psyp70248-bib-0095] O'Connor, M. F. , J. J. Allen , and A. W. Kaszniak . 2002. “Autonomic and Emotion Regulation in Bereavement and Depression.” Journal of Psychosomatic Research 52, no. 4: 183–185. 10.1016/s0022-3999(02)00292-1. 11943236

[psyp70248-bib-0096] O'Connor, M. F. , D. K. Wellisch , A. L. Stanton , R. Olmstead , and M. R. Irwin . 2012. “Diurnal Cortisol in Complicated and Non‐Complicated Grief: Slope Differences Across the Day.” Psychoneuroendocrinology 37, no. 5: 725–728. 10.1016/j.psyneuen.2011.08.009. 21925795 PMC3258306

[psyp70248-bib-0094] O'Connor, M.‐F. 2019. “Grief: A Brief History of Research on How Body, Mind, and Brain Adapt.” Psychosomatic Medicine 81, no. 8: 731–738. 10.1097/PSY.0000000000000717. 31180982 PMC6844541

[psyp70248-bib-0097] Ojha, N. , and A. S. Dhamoon . 2024. Myocardial Infarction. StatPearls. https://www.ncbi.nlm.nih.gov/pubmed/30725761.

[psyp70248-bib-0098] Palitsky, R. , D. T. Wilson , S. E. Friedman , J. M. Ruiz , D. Sullivan , and M. F. O'Connor . 2023. “The Relationship of Prolonged Grief Disorder Symptoms With Hemodynamic Response to Grief Recall Among Bereaved Adults.” Psychosomatic Medicine 85, no. 6: 545–550. 10.1097/psy.0000000000001223. 37260255 PMC10332648

[psyp70248-bib-0099] Panksepp, J. 2003. “Feeling the Pain of Social Loss.” Science 302, no. 5643: 237–239. 10.1126/science.1091062. 14551424

[psyp70248-bib-0100] Paoletti, J. , M. A. Chen , E. L. Wu‐Chung , et al. 2023. “Employment and Family Income in Psychological and Immune Outcomes During Bereavement.” Psychoneuroendocrinology 150: 106024. 10.1016/j.psyneuen.2023.106024. 36702040 PMC9974808

[psyp70248-bib-0101] Persson, S. , A. Warghoff , E. L. Einberg , and P. Garmy . 2021. “Schoolchildren's Experience of Pain—A Focus Group Interview Study.” Acta Paediatrica 110, no. 3: 909–913. 10.1111/apa.15493. 32716530

[psyp70248-bib-0102] Pluess, M. , A. Conrad , and F. H. Wilhelm . 2009. “Muscle Tension in Generalized Anxiety Disorder: A Critical Review of the Literature.” Journal of Anxiety Disorders 23, no. 1: 1–11. 10.1016/j.janxdis.2008.03.016. 18472245

[psyp70248-bib-0103] Poller, W. C. , J. Downey , A. A. Mooslechner , et al. 2022. “Brain Motor and Fear Circuits Regulate Leukocytes During Acute Stress.” Nature 607, no. 7919: 578–584. 10.1038/s41586-022-04890-z. 35636458 PMC9798885

[psyp70248-bib-0104] Pourmohammadi, S. , and A. Maleki . 2020. “Stress Detection Using ECG and EMG Signals: A Comprehensive Study.” Computer Methods and Programs in Biomedicine 193: 105482. 10.1016/j.cmpb.2020.105482. 32408236

[psyp70248-bib-0105] Princip, M. , R. E. Langraf‐Meister , G. M. Slavich , et al. 2022. “Psychosocial and Clinical Characteristics of a Patient With Takotsubo Syndrome and Her Healthy Monozygotic Twin: A Case Report.” European Heart Journal ‐ Case Reports 6, no. 7: ytac255. 10.1093/ehjcr/ytac255. 35832436 PMC9273511

[psyp70248-bib-0106] Raja, S. N. , D. B. Carr , M. Cohen , et al. 2020. “The Revised International Association for the Study of Pain Definition of Pain: Concepts, Challenges, and Compromises.” Pain 161, no. 9: 1976–1982. 10.1097/j.pain.0000000000001939. 32694387 PMC7680716

[psyp70248-bib-0107] Richardson, V. E. , K. M. Bennett , D. Carr , S. Gallagher , J. Kim , and N. Fields . 2015. “How Does Bereavement Get Under the Skin? The Effects of Late‐Life Spousal Loss on Cortisol Levels.” Journals of Gerontology. Series B, Psychological Sciences and Social Sciences 70, no. 3: 341–347. 10.1093/geronb/gbt116. 24259378

[psyp70248-bib-0108] Ridker, P. M. , M. Cushman , M. J. Stampfer , R. P. Tracy , and C. H. Hennekens . 1997. “Inflammation, Aspirin, and the Risk of Cardiovascular Disease in Apparently Healthy Men.” New England Journal of Medicine 336, no. 14: 973–979. 10.1056/NEJM199704033361401. 9077376

[psyp70248-bib-0109] Rodgers, B. L. 2000. Concept Development in Nursing: Foundations, Tech‐Niques, and Applications. Saunders.

[psyp70248-bib-0110] Rona, G. 1985. “Catecholamine Cardiotoxicity.” Journal of Molecular and Cellular Cardiology 17, no. 4: 291–306. 10.1016/s0022-2828(85)80130-9. 3894676

[psyp70248-bib-0111] Rostila, M. , J. Saarela , and I. Kawachi . 2013a. “Fatal Stroke After the Death of a Sibling: A Nationwide Follow‐Up Study From Sweden.” PLoS One 8, no. 2: e56994. 10.1371/journal.pone.0056994. 23451131 PMC3579925

[psyp70248-bib-0112] Rostila, M. , J. Saarela , and I. Kawachi . 2013b. “Mortality From Myocardial Infarction After the Death of a Sibling: A Nationwide Follow‐Up Study From Sweden.” Journal of the American Heart Association 2, no. 2: e000046. 10.1161/JAHA.112.000046. 23537803 PMC3647267

[psyp70248-bib-0113] Rostila, M. , J. Saarela , I. Kawachi , and A. Hjern . 2015. “Testing the Anniversary Reaction: Causal Effects of Bereavement in a Nationwide Follow‐Up Study From Sweden.” European Journal of Epidemiology 30, no. 3: 239–247. 10.1007/s10654-015-9989-5. 25595319

[psyp70248-bib-0114] Roy, R. 1986. “A Psychosocial Perspective on Chronic Pain and Depression in the Elderly.” Social Work in Health Care 12, no. 2: 27–36. 10.1300/j010v12n02_03. 3616890

[psyp70248-bib-0115] Saavedra Pérez, H. C. , N. Direk , J. Milic , M. A. Ikram , A. Hofman , and H. Tiemeier . 2017. “The Impact of Complicated Grief on Diurnal Cortisol Levels Two Years After Loss: A Population‐Based Study.” Psychosomatic Medicine 79, no. 4: 426–433. 10.1097/psy.0000000000000422. 27879552

[psyp70248-bib-0116] Sato, H. , H. Tateishi , T. Uchida , et al. 1990. “Clinical Aspect of Myocardial Injury: From Ischemia to Heart Failure.” Kagaku Hyoronsha 2: 55–64.

[psyp70248-bib-0117] Scally, C. , H. Abbas , T. Ahearn , et al. 2019. “Myocardial and Systemic Inflammation in Acute Stress‐Induced (Takotsubo) Cardiomyopathy.” Circulation 139, no. 13: 1581–1592. 10.1161/CIRCULATIONAHA.118.037975. 30586731 PMC6438459

[psyp70248-bib-0118] Scanzano, A. , and M. Cosentino . 2015. “Adrenergic Regulation of Innate Immunity: A Review.” Frontiers in Pharmacology 6: 171. 10.3389/fphar.2015.00171. 26321956 PMC4534859

[psyp70248-bib-0119] Schafer, S. K. , M. Supke , C. Kausmann , L. M. Schaubruch , K. Lieb , and C. Cohrdes . 2024. “A Systematic Review of Individual, Social, and Societal Resilience Factors in Response to Societal Challenges and Crises.” Community Psychology 2, no. 1: 92. 10.1038/s44271-024-00138-w. PMC11455977 39369098

[psyp70248-bib-0120] Segerstrom, S. C. , and G. E. Miller . 2004. “Psychological Stress and the Human Immune System: A Meta‐Analytic Study of 30 Years of Inquiry.” Psychological Bulletin 130, no. 4: 601–630. 10.1037/0033-2909.130.4.601. 15250815 PMC1361287

[psyp70248-bib-0121] Seiler, A. , K. W. Murdock , and C. P. Fagundes . 2018. “Impaired Mental Health and Low‐Grade Inflammation Among Fatigued Bereaved Individuals.” Journal of Psychosomatic Research 112: 40–46. 10.1016/j.jpsychores.2018.06.010. 30097134

[psyp70248-bib-0122] Seiler, A. , R. von Känel , and G. M. Slavich . 2020. “The Psychobiology of Bereavement and Health: A Conceptual Review From the Perspective of Social Signal Transduction Theory of Depression.” Frontiers in Psychiatry 11: 565239. 10.3389/fpsyt.2020.565239. 33343412 PMC7744468

[psyp70248-bib-0123] Sethi, Y. , H. Murli , O. Kaiwan , et al. 2023. “Broken Heart Syndrome: Evolving Molecular Mechanisms and Principles of Management.” Journal of Clinical Medicine 12, no. 1: 125. 10.3390/jcm12010125. PMC9821117 36614928

[psyp70248-bib-0124] Shonkoff, J. P. , and A. S. Garner . 2012. “The Lifelong Effects of Early Childhood Adversity and Toxic Stress.” Pediatrics 129, no. 1: e232–e246. 10.1542/peds.2011-2663. 22201156

[psyp70248-bib-0125] Singh, T. , H. Khan , M. Mb Bch Bao , et al. 2022. “Takotsubo Syndrome: Pathophysiology, Emerging Concepts, and Clinical Implications.” Circulation 145, no. 13: 1002–1019. 10.1161/CIRCULATIONAHA.121.055854. 35344411 PMC7612566

[psyp70248-bib-0126] Slavich, G. M. 2015. “Understanding Inflammation, Its Regulation, and Relevance for Health: A Top Scientific and Public Priority.” Brain, Behavior, and Immunity 45: 13–14. 10.1016/j.bbi.2014.10.012. 25449576 PMC4361086

[psyp70248-bib-0127] Slavich, G. M. 2016. “Life Stress and Health: A Review of Conceptual Issues and Recent Findings.” Teaching of Psychology 43, no. 4: 346–355. 10.1177/0098628316662768. 27761055 PMC5066570

[psyp70248-bib-0128] Slavich, G. M. 2020a. “Psychoneuroimmunology of Stress and Mental Health.” In The Oxford Handbook of Stress and Mental Health, edited by K. L. Harkness and E. P. Hayden , 519–546. Oxford University Press. 10.1093/oxfordhb/9780190681777.013.24.

[psyp70248-bib-0129] Slavich, G. M. 2020b. “Social Safety Theory: A Biologically Based Evolutionary Perspective on Life Stress, Health, and Behavior.” Annual Review of Clinical Psychology 16: 265–295. 10.1146/annurev-clinpsy-032816-045159. PMC7213777 32141764

[psyp70248-bib-0130] Slavich, G. M. 2022. “Social Safety Theory: Understanding Social Stress, Disease Risk, Resilience, and Behavior During the COVID‐19 Pandemic and Beyond.” Current Opinion in Psychology 45: 101299. 10.1016/j.copsyc.2022.101299. 35219156 PMC8769662

[psyp70248-bib-0131] Slavich, G. M. , and M. R. Irwin . 2014. “From Stress to Inflammation and Major Depressive Disorder: A Social Signal Transduction Theory of Depression.” Psychological Bulletin 140, no. 3: 774–815. 10.1037/a0035302. 24417575 PMC4006295

[psyp70248-bib-0132] Slavich, G. M. , L. G. Roos , S. Mengelkoch , et al. 2023. “Social Safety Theory: Conceptual Foundation, Underlying Mechanisms, and Future Directions.” Health Psychology Review 17, no. 1: 5–59. 10.1080/17437199.2023.2171900. 36718584 PMC10161928

[psyp70248-bib-0133] Slavich, G. M. , and J. Sacher . 2019. “Stress, Sex Hormones, Inflammation, and Major Depressive Disorder: Extending Social Signal Transduction Theory of Depression to Account for Sex Differences in Mood Disorders.” Psychopharmacology 236, no. 10: 3063–3079. 10.1007/s00213-019-05326-9. 31359117 PMC6821593

[psyp70248-bib-0134] Sloth, M. M. B. , J. Hruza , L. H. Mortensen , S. Bhatt , and A. Katsiferis . 2025. “Cause‐Specific Mortality After Spousal Bereavement in a Danish Register‐Based Cohort.” Scientific Reports 15, no. 1: 6240. 10.1038/s41598-025-90657-1. 39979402 PMC11842573

[psyp70248-bib-0135] Spillane, A. , K. Matvienko‐Sikar , C. Larkin , P. Corcoran , and E. Arensman . 2018. “What Are the Physical and Psychological Health Effects of Suicide Bereavement on Family Members? An Observational and Interview Mixed‐Methods Study in Ireland.” BMJ Open 8, no. 1: e019472. 10.1136/bmjopen-2017-019472. PMC5781012 29331974

[psyp70248-bib-0136] Stroebe, M. , H. Schut , and W. Stroebe . 2007. “Health Outcomes of Bereavement.” Lancet 370, no. 9603: 1960–1973. 10.1016/S0140-6736(07)61816-9. 18068517

[psyp70248-bib-0137] Tawakol, A. , A. Ishai , R. A. Takx , et al. 2017. “Relation Between Resting Amygdalar Activity and Cardiovascular Events: A Longitudinal and Cohort Study.” Lancet 389, no. 10071: 834–845. 10.1016/S0140-6736(16)31714-7. 28088338 PMC7864285

[psyp70248-bib-0138] Tegegne, B. S. , M. A. Said , A. Ani , et al. 2023. “Phenotypic but Not Genetically Predicted Heart Rate Variability Associated With All‐Cause Mortality.” Communications Biology 6, no. 1: 1013. 10.1038/s42003-023-05376-y. 37803156 PMC10558565

[psyp70248-bib-0139] Templin, C. , J. R. Ghadri , J. Diekmann , et al. 2015. “Clinical Features and Outcomes of Takotsubo (Stress) Cardiomyopathy.” New England Journal of Medicine 373, no. 10: 929–938. 10.1056/NEJMoa1406761. 26332547

[psyp70248-bib-0140] Thayer, J. F. , and R. D. Lane . 2000. “A Model of Neurovisceral Integration in Emotion Regulation and Dysregulation.” Journal of Affective Disorders 61, no. 3: 201–216. 10.1016/s0165-0327(00)00338-4. 11163422

[psyp70248-bib-0141] Thimm, J. C. , A. E. Kristoffersen , and U. Ringberg . 2020. “The Prevalence of Severe Grief Reactions After Bereavement and Their Associations With Mental Health, Physical Health, and Health Service Utilization: A Population‐Based Study.” European Journal of Psychotraumatology 11, no. 1: 1844440. 10.1080/20008198.2020.1844440. 33408813 PMC7748058

[psyp70248-bib-0142] Tofler, G. H. , M. C. Morel‐Kopp , M. Spinaze , et al. 2020. “The Effect of Metoprolol and Aspirin on Cardiovascular Risk in Bereavement: A Randomized Controlled Trial.” American Heart Journal 220: 264–272. 10.1016/j.ahj.2019.11.003. 31923768

[psyp70248-bib-0143] Tofthagen, R. , and L. M. Fagerstrom . 2010. “Rodgers' Evolutionary Concept Analysis—A Valid Method for Developing Knowledge in Nursing Science.” Scandinavian Journal of Caring Sciences 24, no. Suppl 1: 21–31. 10.1111/j.1471-6712.2010.00845.x. 21070310

[psyp70248-bib-0144] Tsigos, C. , and G. P. Chrousos . 2002. “Hypothalamic‐Pituitary‐Adrenal Axis, Neuroendocrine Factors and Stress.” Journal of Psychosomatic Research 53, no. 4: 865–871. 10.1016/s0022-3999(02)00429-4. 12377295

[psyp70248-bib-0145] Van den Bergh, O. , M. Witthoft , S. Petersen , and R. J. Brown . 2017. “Symptoms and the Body: Taking the Inferential Leap.” Neuroscience and Biobehavioral Reviews 74, no. Pt A: 185–203. 10.1016/j.neubiorev.2017.01.015. 28108416

[psyp70248-bib-0146] Vilela, E. M. , and R. Fontes‐Carvalho . 2021. “Inflammation and Ischemic Heart Disease: The Next Therapeutic Target?” Revista Portuguesa de Cardiologia 40: 785–796. 10.1016/j.repc.2021.02.011. 34857118

[psyp70248-bib-0147] Wei, D. , I. Janszky , F. Fang , et al. 2021. “Death of an Offspring and Parental Risk of Ischemic Heart Diseases: A Population‐Based Cohort Study.” PLoS Medicine 18, no. 9: e1003790. 10.1371/journal.pmed.1003790. 34587153 PMC8480908

[psyp70248-bib-0148] Wei, D. , I. Janszky , J. Li , and K. D. László . 2023. “Loss of a Child and the Risk of Atrial Fibrillation: A Danish Population‐Based Prospective Cohort Study.” Journal of Epidemiology and Community Health 77, no. 5: 322–327. 10.1136/jech-2022-219695. 36858813 PMC10086482

[psyp70248-bib-0150] Wei, D. , I. Janszky , R. Ljung , et al. 2021. “Bereavement in the Year Before a First Myocardial Infarction: Impact on Prognosis.” European Journal of Preventive Cardiology 28, no. 11: 1229–1234. 10.1177/2047487320916958. 34551078

[psyp70248-bib-0149] Wei, D. , I. Janszky , R. Ljung , F. Fang , J. Li , and K. D. László . 2022. “Bereavement and Prognosis After a First Acute Myocardial Infarction: A Swedish Register‐Based Cohort Study.” Journal of the American Heart Association 11, no. 17: e027143. 10.1161/jaha.122.027143. 36056733 PMC9496408

[psyp70248-bib-0151] Wei, D. , J. Li , H. Chen , et al. 2022. “Death of a Child and the Risk of Stroke: A Binational Cohort Study From Denmark and Sweden.” Neurology 98, no. 11: e1104–e1113. 10.1212/wnl.0000000000013263. 34996877

[psyp70248-bib-0152] Wei, D. , J. Li , I. Janszky , et al. 2022. “Death of a Child and the Risk of Heart Failure: A Population‐Based Cohort Study From Denmark and Sweden.” European Journal of Heart Failure 24, no. 1: 181–189. 10.1002/ejhf.2372. 34693593

[psyp70248-bib-0153] Wei, D. , T. Olofsson , H. Chen , et al. 2021. “Death of a Child and the Risk of Atrial Fibrillation: A Nationwide Cohort Study in Sweden.” European Heart Journal 42, no. 15: 1489–1495. 10.1093/eurheartj/ehaa1084. 33515041 PMC8046501

[psyp70248-bib-0154] Wijesurendra, R. S. , and B. Casadei . 2019. “Mechanisms of Atrial Fibrillation.” Heart (British Cardiac Society) 105, no. 24: 1860–1867. 10.1136/heartjnl-2018-314267. 31444267

[psyp70248-bib-0155] Wilhelm, F. H. , M. C. Pfaltz , P. Grossman , and W. T. Roth . 2006. “Distinguishing Emotional From Physical Activation in Ambulatory Psychophysiological Monitoring.” Biomedical Sciences Instrumentation 42: 458–463. 16817651

[psyp70248-bib-0156] Wilhelm, F. H. , J. A. Rattel , M. Wegerer , et al. 2017. “Attend or Defend? Sex Differences in Behavioral, Autonomic, and Respiratory Response Patterns to Emotion‐Eliciting Films.” Biological Psychology 130: 30–40. 10.1016/j.biopsycho.2017.10.006. 29054817

[psyp70248-bib-0157] Willeit, P. , A. Thompson , T. Aspelund , et al. 2013. “Hemostatic Factors and Risk of Coronary Heart Disease in General Populations: New Prospective Study and Updated Meta‐Analyses.” PLoS One 8, no. 2: e55175. 10.1371/journal.pone.0055175. 23408959 PMC3567058

[psyp70248-bib-0158] Wittstein, I. S. , D. R. Thiemann , J. A. Lima , et al. 2005. “Neurohumoral Features of Myocardial Stunning due to Sudden Emotional Stress.” New England Journal of Medicine 352, no. 6: 539–548. 10.1056/NEJMoa043046. 15703419

[psyp70248-bib-0159] Wu, E. L. , A. S. LeRoy , C. J. Heijnen , and C. P. Fagundes . 2021. “Inflammation and Future Depressive Symptoms Among Recently Bereaved Spouses.” Psychoneuroendocrinology 128: 105206. 10.1016/j.psyneuen.2021.105206. 33866069 PMC13235533

[psyp70248-bib-0160] Wu‐Chung, E. L. , R. L. Brown , R. Suchting , et al. 2025. “Spousal Bereavement Enhances Proinflammatory Cytokine Production to Acute, Psychological Stress.” Psychoneuroendocrinology 178: 107498. 10.1016/j.psyneuen.2025.107498. 40435540 PMC13383667

[psyp70248-bib-0161] Wu‐Chung, E. L. , K. M. Kennedy , L. D. Medina , et al. 2025. “Cortical Thickness and Low‐Grade Inflammation Moderate the Association Between Depressive Symptoms and Cognitive Function in Early Widowhood: A Preliminary Study.” Brain, Behavior, and Immunity 129: 620–633. 10.1016/j.bbi.2025.06.027. 40541668 PMC13472649

[psyp70248-bib-0162] Young, E. , and A. Korszun . 2010. “Sex, Trauma, Stress Hormones and Depression.” Molecular Psychiatry 15, no. 1: 23–28. 10.1038/mp.2009.94. 19773810

[psyp70248-bib-0163] Zhang, J. M. , and J. An . 2007. “Cytokines, Inflammation, and Pain.” International Anesthesiology Clinics 45, no. 2: 27–37. 10.1097/AIA.0b013e318034194e. 17426506 PMC2785020

